# Reinforcing bitumen with highly graphitized reduced graphene oxide derived from coal Tar pitch via template free sole step hydrothermal strategy

**DOI:** 10.1038/s41598-025-26992-0

**Published:** 2025-11-24

**Authors:** Z. L. Abo-Shanab, Eslam A. Mohamed, Marwa Adel

**Affiliations:** 1 Petroleum Applications Department, Egyptian Petroleum Research Institute, P.O. 11776, Nasr City, Cairo Egypt; 2 Fabrication Technology Department Advanced Technology and New Materials Research Institute, City of Scientific Research and Technological Applications (SRTA City), New Borg El- Arab, Alexandria, 21934 Egypt

**Keywords:** Bitumen, Graphene, Reduced graphene, Coal tar, Marshall, Rutting, Chemistry, Energy science and technology, Engineering, Environmental sciences, Materials science

## Abstract

This study pioneers a cost-effective and scalable technique for preparing high-quality, highly-graphitized reduced graphene oxide (HG-RGO) from coal tar pitch (CTP), as an abundant aromatic precursor. The novelty lies in developing a one-pot, hard template-free hydrothermal method utilizing cetyltriethyl ammonium bromide (CTAB) at a moderate temperature, 270 °C to facilitate the formation of the desired nanostructure. Comprehensive microstructural analysis, utilizing XRD, FTIR, Raman spectroscopy, HR-TEM, and SAED patterns, confirmed the successful preparation of HG-RGO. This HG-RGO was then utilized to modify bitumen (penetration grade 80/100) at varying concentrations (1 wt%, 3 wt%, and 5 wt%) to create graphene-modified bitumen (GMB). The study meticulously assessed the impact of HG-RGO on the physical and mechanical properties of both the bitumen binder and the resulting asphalt mixtures. The results unequivocally demonstrate that HG-RGO acts as a potent modifier, effectively upgrading the soft bitumen pen grade 80/100 to the harder pen grade 60/70. Key physical property improvements were quantified: the Softening Point increased by 10 to 55 °C, the Penetration degree decreased by 17 to 68dmm, and Kinematic Viscosity increased by 200cSt to 550cSt.Furthermore, the HG-RGO significantly enhances the mechanical characteristics of the asphalt mixtures. The Marshall Stability reached a maximum of 2200 kg for the 5wt% GMB mixture, and the rutting depth decreased threefold compared to the unmodified binder, reaching just 5 mm for GMB5. Dynamic Mechanical Analyzer (DMA) results confirmed improved rutting resistance at high temperatures and enhanced fatigue performance, evidenced by decreased loss modulus. These findings suggest that HG-RGO can be successfully utilized as a modifier for soft bitumen for pavements like traditional bitumen pen grade 60/70.

## Introduction

Coal tar pitch (CTP), a by-product of the coal tar distillation process, has long been utilized across various chemical and manufacturing industries. In recent years, CTP has also attracted attention as a promising precursor for developing new carbon materials^1–4^, owing to its high production volume, low cost, and rich aromaticity. However, commercially available CTP faces notable challenges, including a relatively low carbon yield compared to its carbon content and a high level of quinoline-insoluble (QI) fractions, which can introduce defects into the resulting carbon materials. Consequently, various technical treatments have been explored to modify CTP and enhance its properties for more advanced applications. Extensive research has focused on improving the properties of coal tar pitch (CTP) through additives, with numerous studies reporting treatment methods that enhance its carbonization behavior^6^. Arani et al.^6^ investigates how the addition of carbon nanotubes (CNTs) and carbon black (CB) affects the graphitization of coal tar pitch (CTP), aiming to evaluate their influence on enhancing the graphitization degree. This study neglects the cost concerns where CNTs are expensive, which could limit the practical, large-scale application of the findings in industry. Although many studies are employed to achieve higher graphitization, they disregard the energy and process intensity. They frequently rely on applied high temperatures exceeding 3000 °C or lengthy treatments, more than 20 h, that may not be energy-efficient for industrial adoption^6,7^.

CTP is rich in heterocyclic compounds and polycyclic aromatic hydrocarbons featuring sp^2^-hybridized carbon atoms, closely resembling the hexagonally arranged carbon structure of graphene; thus, it can be regarded as small-sized graphene to some degree. Liquefied pitch prefers to be coated onto the highly-dispersed template surface, resulting in a thin coating layer^8^. These aromatic molecules can undergo polymerization and further aromatization within a layered template-confinement nano space, forming large interconnected thin polymeric films. Upon high-temperature treatment, these films can subsequently be transformed into graphene nanosheets within the confined environment. X. He et al. employed layered-template nanospace confinement and in situ chemical activation to synthesize carbon graphene nanosheets (CGNS) from petroleum pitch. The resulting CGNS feature hierarchical porosity and large sheet structures, heating up to 1073 K at 5 K min^− 1^ and kept for one hour to make CGNSs under an inert atmosphere. The CGNSs were obtained by acid washing with 2 M HCl solution and distilled water repeatedly to remove the template and inorganic impurities. However, the high amounts of costly soft templates and the complex post-treatment processes required for their removal limit the large-scale synthesis of such graphitic material for practical applications. Therefore, it is crucial to develop a hard template-free approach for their production. Thus, herein this study developed one-pot hard template-free hydrothermal treatment of coal-tar pitch at moderate temperature 270 °C, by employing CTAB (CetylTriethyl Ammonium Bromide) and a few millilitres of ammonium hydroxide solution not as a hard or constant structural template, but somewhat as a dispersing agent and temporary surfactant that facilitates the self-assembly of graphitic domains and exfoliation process during hydrothermal treatment. A deep microstructure assessment for the as-prepared graphitic material was conducted using XRD, FTIR, Raman spectroscopy, HR-TEM, and SAED patterns.

Nanomaterials are gaining significant attention in the asphalt industry due to their unique characteristics^10,11^. Their tiny size, typically 1 to 100 nm, gives them an exceptionally high specific surface area. This property is crucial, allowing them to interact extensively with the bitumen matrix, improving performance^12^. Researchers have explored various nanomaterials for bitumen modification, including carbon nanotubes, nano-clay^13^, nano-silicon dioxide^14^, nano fly ash^15^, carbon nanofibers^16^, and nano titanium dioxide^17^. Among these, graphene and its derivatives have garnered exceptional interest. This is mainly because of their outstanding mechanical, thermal, and electrical properties^18^. The high mechanical strength of graphene, for instance, allows it to act as a powerful reinforcing agent within the bitumen, significantly increasing its stiffness and resistance to deformation^19^.

Furthermore, graphene’s high specific surface area enables it to form strong interactions with the bitumen’s molecular components, creating a more stable and robust composite material^20^. This has made graphene-based materials an increasingly popular choice for bitumen modification, moving beyond traditional polymer additives to achieve good asphalt performance^21^. The ability of graphene to enhance bitumen’s properties at a molecular level makes it a promising material for developing more durable and long-lasting asphalt pavements^22^. The primary novelty in this study was the upcycling of coal-tar pitch, which has a low-cost, abundant industrial waste product into a high-value material. This provides a sustainable and economically viable alternative to expensive, conventional graphene precursors. The use of a template-free, one-pot hydrothermal route further adds to this novelty, as it is a simple, green, and scalable method that avoids harsh chemicals and complex multi-step processes work is not simply the preparation of graphene but the creation of Highly Graphitized Reduced Graphene Oxide (HG-RGO) with fascinating micro-structure characteristics without the need to costly soft templates and the complex post-treatment processes required for their removal. This unique, high-quality structure is critical since it directly affects the performance of bitumen. The ultimate novelty is the application and the results. Our study aimed to replace bitumen pen grade 80/100 with bitumen grade 60/70 by incorporating HG-RGO.

## Experimental

### Materials

Coal tar pitch (CTP; derived from El Nasr for Coke), cetyltriethyl ammonium bromide (CTAB, 99.5%, WINLAB, UK), and ammonium hydroxide (NH₄OH, 33% NH₃, Sigma-Aldrich, Europe) were used as received without further purification, as all chemicals were of analytical grade. Double-distilled water was employed as the solvent throughout the synthesis process. Bitumen 80/100 derived from the Suez Company for oil refinery. Coarse and fine aggregates, dolomite grade derived from “ATAKA” quarry, silica sand derived from “ELREHAB” quarry.

### Synthesis of HG-RGO

In a single, streamlined procedure, an 82 mM aqueous solution of cetyltriethyl ammonium bromide (CTAB) was combined with coal tar pitch (CTP) at a 1:1 volumetric ratio. A small ammonium hydroxide solution was added to this mixture at a 1/10 volumetric ratio to act as a crucial structure-directing agent. The resulting mixture was then carefully sealed within a Teflon-lined stainless-steel autoclave. A vapour-to-liquid volume proportion of 3:1 was maintained to facilitate the hydrothermal reaction. The autoclave was subsequently placed in a furnace and subjected to a precisely controlled thermal treatment at 270 °C for 16 h. This meticulous one-pot process was designed to effectively synthesize highly graphitized reduced graphene oxide (HG-RGO) from the coal tar pitch precursor.

### Characterization of HG-RGO

X-ray diffraction (XRD) was employed to identify and characterize the crystalline structure of the synthesized powder sample. XRD patterns were recorded using a Shimadzu XRD-7000 diffractometer (30 kV, 30 mA, Cu Kα radiation with a Ni filter, λ = 0.15406 nm; Shimadzu Corporation, Tokyo, Japan). The analysis was performed over two hours with a 4°/min scanning rate and a step size of 0.02°. Fourier Transform Infrared (FTIR) spectroscopy was conducted to qualitatively assess the oxidation states of the graphitic material using a Shimadzu FTIR-8400 S spectrometer (Shimadzu Corporation, Kyoto, Japan). Spectra were collected across the wavenumber range of 500–4000 cm⁻¹ with a resolution of 5 cm⁻¹ and an average of 25 scans. Raman spectroscopy, a widely recognized nondestructive technique for characterizing carbon nanostructures, was used to investigate the in-plane crystallite size, degree of disorder, and extent of chemical modification. Raman spectra were obtained using a Bruker SENTERRA Raman Microscope (Bruker Corporation) with 532 nm laser excitation over the range of 500–4000 cm^−^¹. High-resolution transmission electron microscopy (HR-TEM) images were captured using a JEM-2100 Plus (LaB_6_ filament, JEOL USA, Inc.), operating at accelerating voltages of 20 and 200 kV, to explore the microstructure of the samples further.

### Bitumen modification by HG-RGO

Preparing high-quality graphene-modified bitumen requires meticulous attention to the mixing conditions to ensure a stable and homogeneous dispersion of the HG-RGO^23^. The key challenge is to prevent graphene from clumping, which would hinder its reinforcing capabilities. Our method addresses this by first dispersing 1 gram of HG-RGO in 5 ml of xylene solvent, leveraging the solvent’s aromatic structure to promote favourable interactions with the graphene sheets. This pre-dispersion step is critical for breaking up graphene agglomerates. The solution is then slowly added to 100 g of bitumen at 155 °C and manually stirred for 5 min. This initial mixing is followed by an hour-long session with a high-speed mixer at 180 °C and 5000 rpm. This intense shear mixing ensures that the HG-RGO is uniformly distributed throughout the bitumen and facilitates the anthracene solvent’s complete evaporation. We prepared three different mixtures by varying the HG-RGO content to 1 wt% (GMB1), 3 wt% (GMB3), and 5 wt% (GMB5), allowing us to study the impact of graphene concentration on the final properties of the bitumen.

### Physical characterization of GMB

The physical properties were studied, including penetration at 25 °C according to ASTM D 5, softening point test according to ASTM D 36, and kinematic viscosity as recommended by ASTM D2170. Then the samples of GMB are subjected to a short-term ageing process using RTFO according to ASTM D2872. An asphalt sample is placed on a thin film oven with a thermostat and a rotating disc rack at 163 °C for five hours. Also, the storage stability of prepared GMB binders during long-term storage at high temperature is examined according to ASTM D 7173^24^.

### Mix grading

The asphaltic wear surfaces mix design (mix 4 C) consists of 12% pin 1, 10% pin 2, 22% pin 3 (coarse aggregate), 50% sand, and 6% limestone dust as the mineral fillers.

Table 1 shows the planned grading of the concrete with asphalt mix, which is beyond the specification limitations of Egyptian standards for wearing surfaces (mix 4 C)^25^.

**Table 1:** Job mix formula (JMF) for surfacing course (4 C).

Sieve	Pin1		Pin 2		Pin 3		Crushed Sand		Dust		JMF	JMT	
	Passing	12%	Passing	10%	Passing	22%	Passing	50%	Passing	6%		Min	Max
1”	100	12	100	10	100	22	100	50	100	6	100	100	100
3/4”	94	11.28	100	10	100	22	100	50	100	6	99.2	80	100
1/2”	15	1.8	64	6.4	87	19.4	100	50	100	6	83.3	.	.
3/8”	2	0.24	20	2	30	6.6	100	50	100	6	64.8	60	70
no. 4	1	0.12	0	0	2	0.44	95	47.5	100	6	54.06	48	65
no 8	0	0	0	0	0	0	75	37.5	100	6	43.5	35	50
no. 16	0	0	0	0	0	0	55	27.5	100	6	33.5	…	…
no. 30	0	0	0	0	0	0	36	18	100	6	24	19	30
no.50	0	0	0	0	0	0	18	9	100	6	15	13	23
no.100	0	0	0	0	0	0	8	4	91	5.4	9.4	7	15
no 200	0	0	0	0	0	0	2.2	1.1	65	3.9	5	3	8

### Dynamic shear rheometer test

The Dynamic Shear Rheometer (DSR) test was performed on both unmodified and HG-RGO modified bitumen using a Triton Technology-TTDMA rheometer in accordance with ASTM D-7175 standards. Samples were prepared by heating the bitumen to 150 °C for two hours, pouring the material into silicon rubber molds to create 8 mm diameter and 2 mm thickness samples, and then allowing them to cool for one hour. To ensure reliable results, samples were loaded at 6 °C below the testing temperature and pre-sheared to eliminate any historical load associated with preparation and handling ^4,26^. The detailed preparation and testing protocols are provided in Table 2, and the subsequent sections summarize the specific temperature and time sweep tests selected to evaluate the bitumen performance.

**Table 2:** Test matrix for DMA-based sweep tests.

Sample Name	Sweep type	Testing Temp [°C]	No of Tests	Strain[%]	Frequency [hz]
GMB	Temperature	25 to70	3	12%	1.59
	Time	35	3	12%	1.59

#### Temperature sweep test

Samples were prepared and trimmed at 25 °C, then subjected to a pre-shearing conditioning period of 30 s at a 12% strain and 1.59 Hz frequency (as detailed in Table 2). The test was performed by collecting dynamic shear rheometer data at 3 °C intervals across a temperature range of 25 °C to 70 °C, maintaining a 3-minute equilibrium time at each data point. The resulting rutting parameter, G^∗^/sin(δ), was plotted against the testing temperature and compared to the Superpave limiting criterion (G^∗^/sin(δ) ≥ 1 kPa) to precisely determine the actual high-performance grade (PG) temperature for the asphalt binder.

#### Time sweep test

Bitumen samples were tested at 35 °C throughout the experiment, beginning with a 5-minute waiting period to achieve thermal equilibrium. As detailed in Table 2, the subsequent 15-minute time sweep test was initiated with a 30-second pre-shearing phase conducted at a strain of 12% and a frequency of 1.59 Hz. Following this, the resulting complex modulus and loss tangent were continuously monitored and plotted as a function of the total loading time.

### Marshall test

**Fig. 1:**
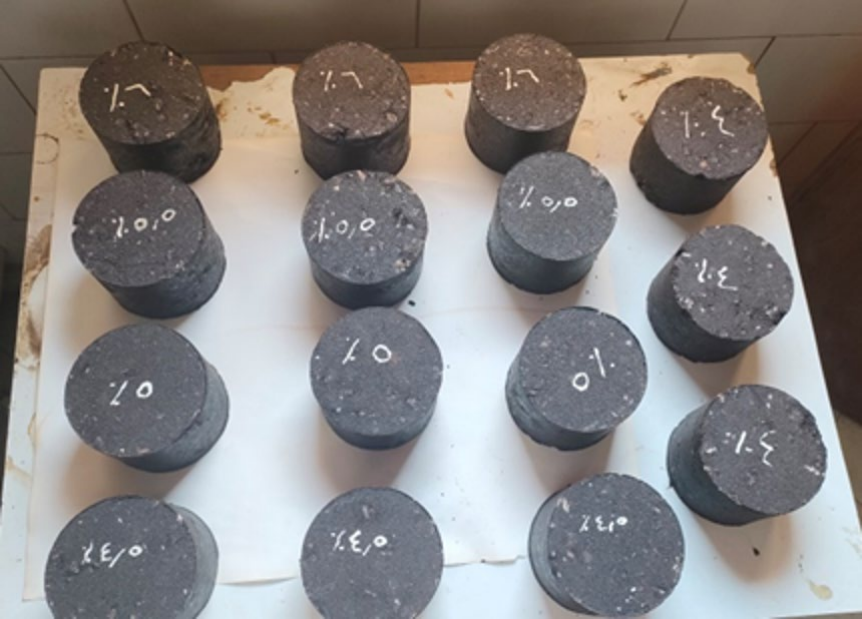
Samples of compacted **GMB** mixture for Marshall Tests.

The resistance of the modified asphalt to plastic flow was comprehensively evaluated using the Marshall Stability and flow test^27^, performed with a Marshall Device model TO-550-1 from the United States. This crucial test determines the stability (in kg), representing the maximum load a compacted asphalt mixture can withstand before plastic deformation, and the flow value (in mm) measures the mixture’s deformation at maximum load^28^. To thoroughly assess the impact of the graphene modification, compacted asphalt samples were prepared for three different graphene-modified bitumen (GMB) percentages: GMB1, GMB3, and GMB5, alongside a control sample (neat bitumen 80/100) for baseline comparison. For each of these GMB binders, five distinct binder content percentages (4 wt%, 4.5 wt%, 5 wt%, 5.5 wt%, and 6 wt%) were investigated. This meticulous variation in binder content allowed for the optimization of the mix design and a detailed understanding of how graphene integration influences the mechanical properties of the asphalt mixture, all conducted strictly with ASTM D 6927 standards^27,29^, as shown in Fig. 1.

### Double wheel tracker (DWT) Hamburg type

**Fig. 2:**
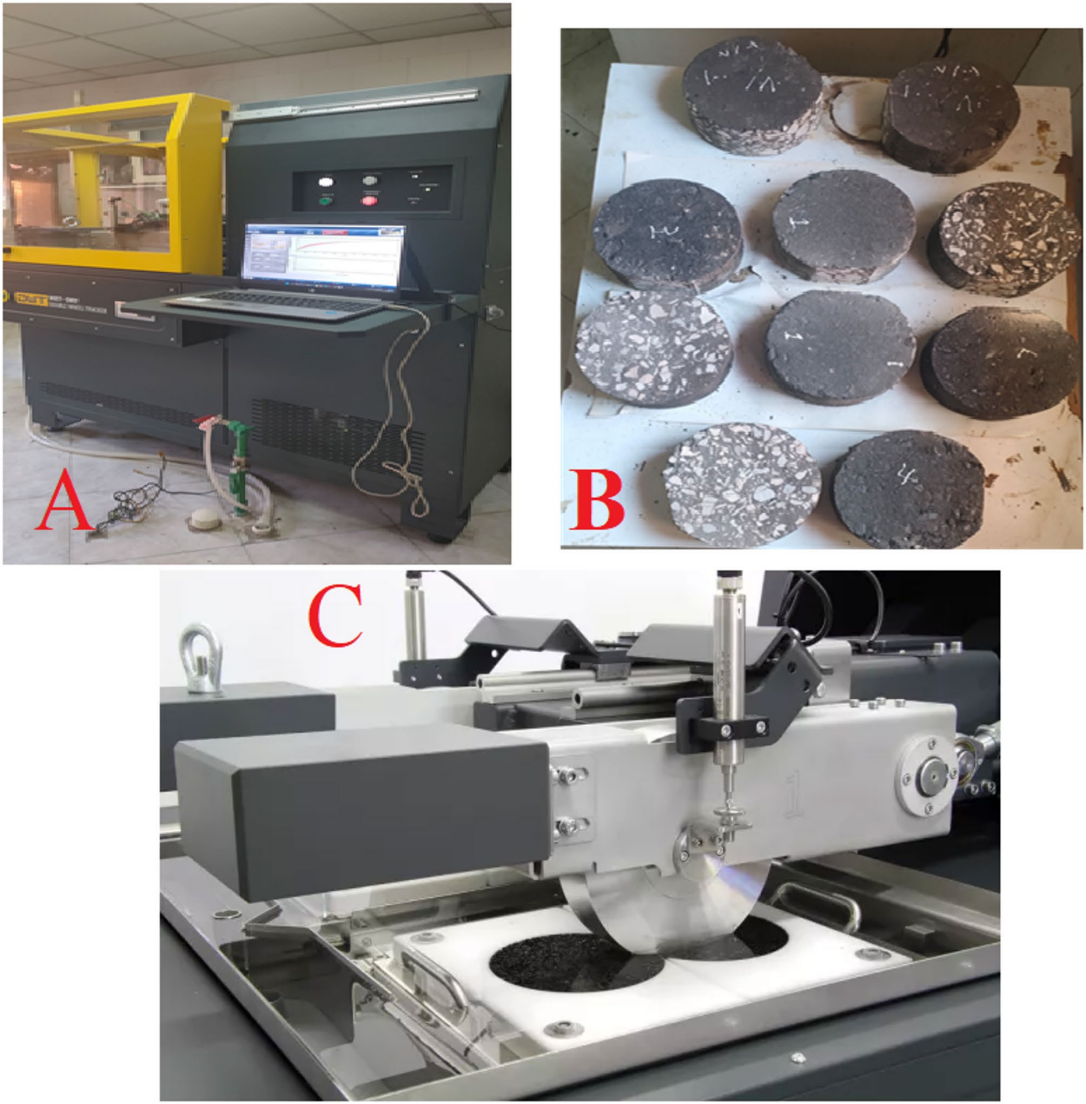
(**A**) Double Wheel Tracker device (DWT), (**B**) Prepared compacted GMB mixtures for rutting test, and (**C**) Two Slabs under a wheel load.

Hot Mix Asphalt (HMA) resistance to permanent deformation, a critical performance indicator known as rutting, was rigorously evaluated using a Double Wheel Tracker (manufactured in Russia, as shown in Fig. 2A). This sophisticated device precisely measures the rut depths created by numerous passes of weighted wheels at a constant, elevated temperature, thereby simulating traffic loading under hot conditions. The test rigorously adheres to recognized international standards, specifically AASHTO T-324 and EN 12697-22, ensuring the reliability and comparability of the results. For each GMB (Graphene Modified Bitumen) mixture, representative asphalt samples were prepared as double cylindrical slabs, meticulously compacted to 150 mm in height and 200 mm in diameter using a Linear Kneading Compactor, in accordance with the LTG 2015 guidelines^30^. These compacted cores were then precisely cut to provide an adequate wheel path width and height for the traversing wheel (Fig. 2B). The experiment was established at a constant temperature of 60 °C, applying a continuous load of 705 N per wheel. Rut depths were continuously monitored and measured with high precision (0.01 mm resolution) by sensors on each wheel, with measurements recorded at 45-minute intervals throughout the test. The detailed setup for this comprehensive evaluation is depicted in Fig. 2C. This methodical approach directly assesses the asphalt mixture’s ability to resist plastic flow under conditions directly relevant to in-service pavement performance^31^.

## Results and discussion

### Characterization of HG-RGO

#### XRD pattern of HG-RGO

**Fig. 3:**
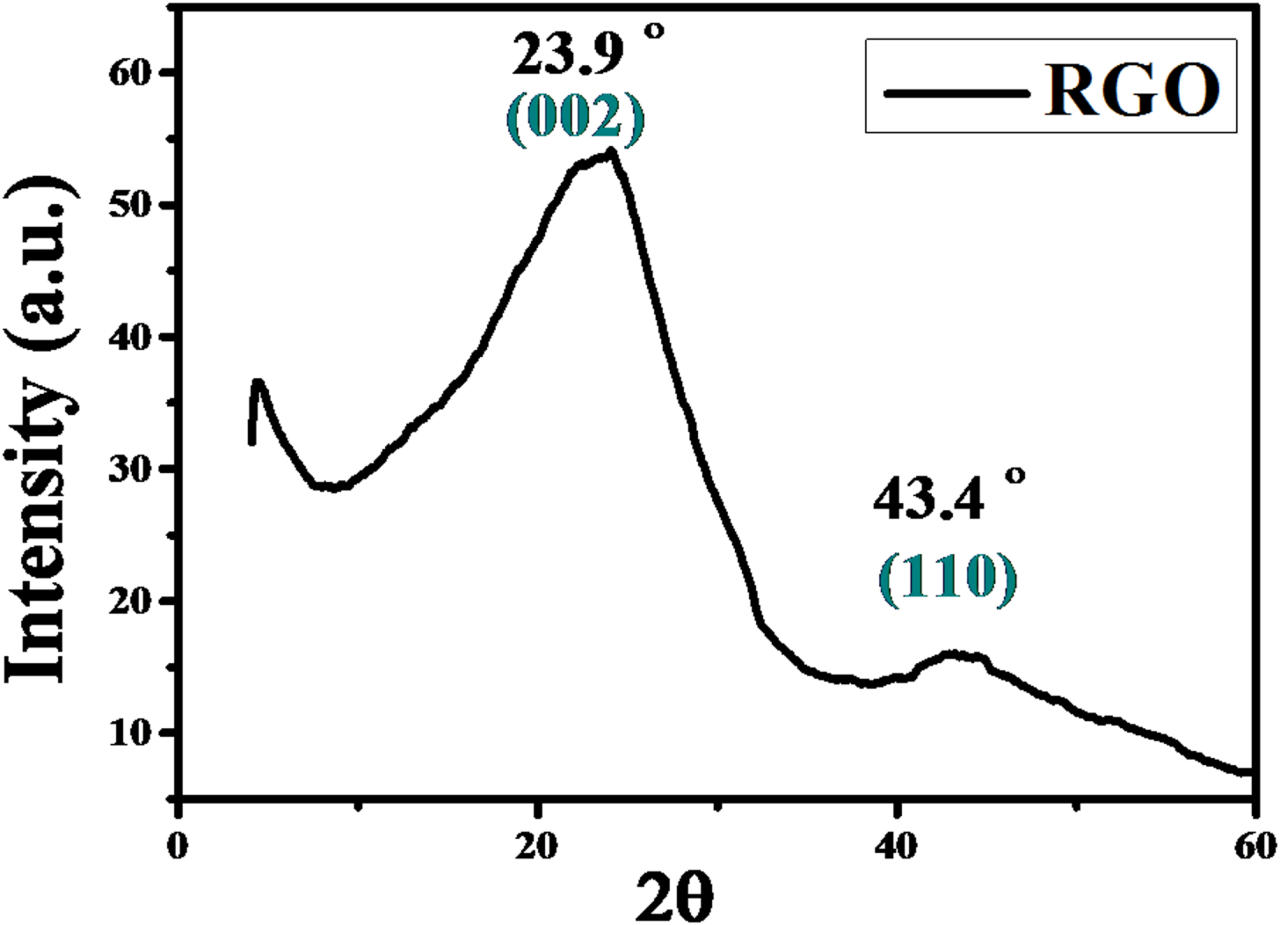
XRD diffraction patterns of the as-prepared highly graphitized reduced graphene oxide HG-RGO.

Figure 3 represents the XRD pattern of the as-synthesized shiny black powdery sample produced at hydrothermal temperature 270 °C, HG-RGO. The appearance of a broad peak between 2θ = 20–30° and centred at 23.9° with interplanar distance 0.38 nm is associated with (002), the graphitic material’s lattice plane. This means the domination of the π-conjugation structure within the produced graphitic material. The peak’s broadness reveals that the crystal phase (002) is randomly arranged, not regularly oriented as in the highly crystalline graphite structure, which is specified with a sharp, intense peak. Hence, this indicates the exfoliated nature of the formed graphitic structure. The appearance of the (110) plane of graphite is indexed near 43 °, indicating a more regular structure of the carbonized products than that of the raw CTP^5^.

The average crystallite size (thickness), perpendicular to the (002) plane, expressing thus the staking height of RGO layers *(D*_*002*_ *or L*_*c*_*)*, has been calculated using Scherrer’s equation, which is expressed by Eq. 1

1 $${{\text{D}}_{00{\text{2}}}}\,=\,{\text{Kl}}/{\text{Bcosq}}$$

where K is a constant reliant on the crystallite shape (0.89), λ is the X-ray wavelength, B is the full width at half maximum, and θ is the scattering angle of the (002) peak. The average number of staked layers (NGL) has been consequently computed via the following Eq. 2

2 $${\text{NGL}}\,=\,{{\text{D}}_{00{\text{2}}}}/{{\text{d}}_{00{\text{2}}}}$$

The average staking height was found to be ~ 1.76 nm. Thereby, NGL values suggest that the samples have an average of 4–5 RGO layers. Notably, the average crystallite size of the as-prepared graphitic sample HG-RGO differs from that of CTP( L_c_ = 14.89 nm, according to the literature^6^.

#### FTIR analysis of HG-RGO

**Fig. 4:**
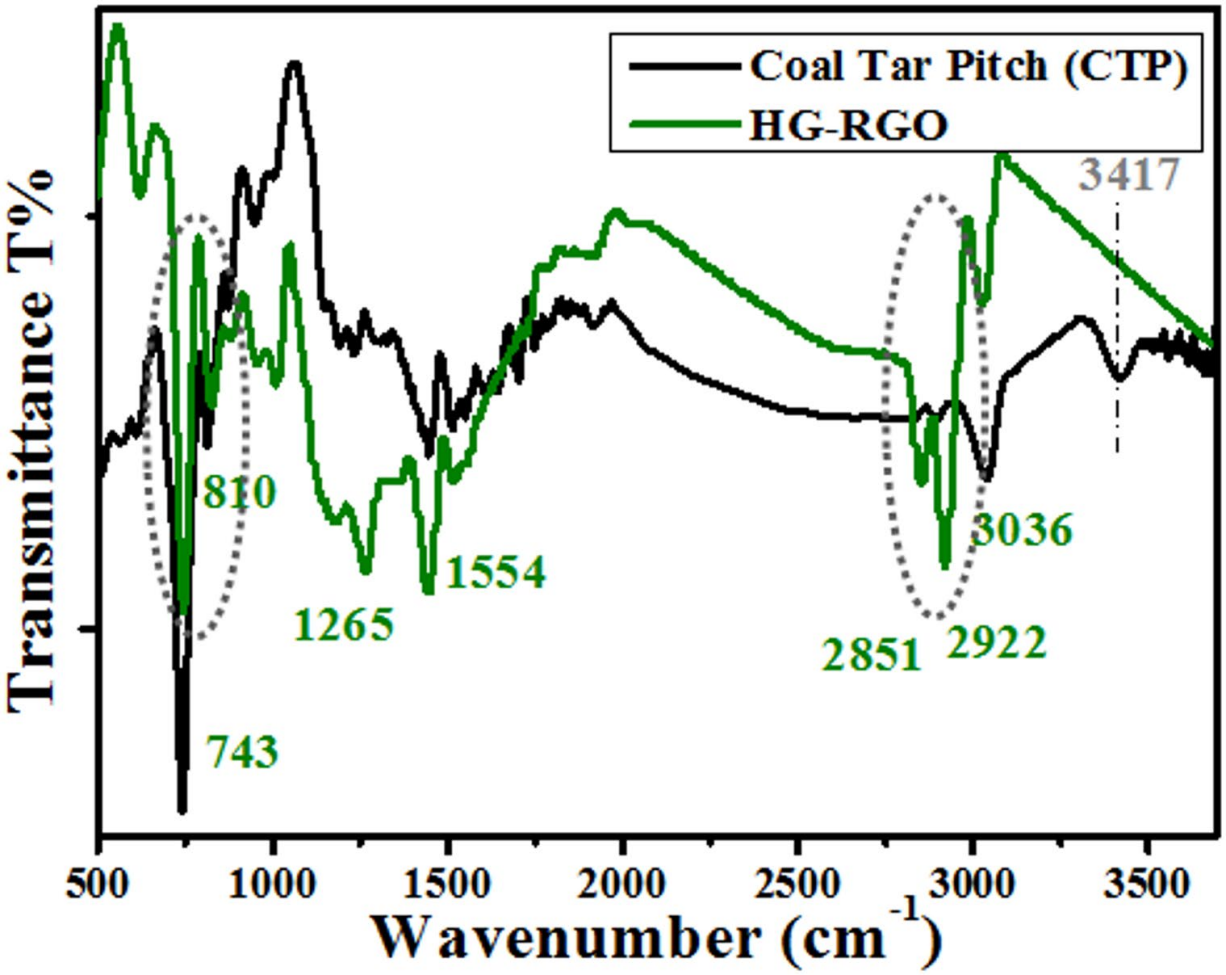
FTIR spectra of coal tar pitch (CTP) and the as-prepared highly graphitized reduced graphene oxide HG-RGO.

Figure 4 depicts the FTIR spectra of the raw CTP and the as-prepared HG-RGO. The peak located at 3036 cm^− 1^ is related to the aliphatic C-H groups. Those two bands at 2922 cm^− 1^and 2851 cm^− 1^ belong to the asymmetric and symmetric stretching vibrations of the methylene groups CH_2_. Those bands appear to be strong bands in the spectrum of the as-prepared HG-RGO compared with the ones of the raw CTP. This indicates the graphitic structure of the HG-RGO is functionalized with CH_2_ from the main chain of the CTAB molecules. The aromaticity is indicated from the appearance of the absorption band at around 1554 cm^− 1^, which is attributed to the in-plane C = C stretching vibration. The two peaks at 1509 cm^− 1^ and 1265 cm^− 1^ are related to the plane bending vibrations of C-H. The appearance of the absorption peaks around 810 –743 cm^− 1^ corresponds to C-H out-of-plane bending vibrations and stretching vibrations of the aromatic structures. Those two peaks are becoming less intense in the spectrum of HG-RGO than those of the raw CTP. This implies that the hydrogen removed during the carbonization/graphitization process is aromatic and predominantly comes from the aromatic rings. This suggests there are multi-fused rings formed through the polymerization process during the graphitization process of RGO^5,32^.

The strong absorption peak at 3417 cm^− 1^, which appears in the spectrum of CTP, is related to H_2_O molecules. The reduced nature of the as-prepared graphitic material is confirmed by the complete disappearance of this band in its FTIR spectrum. This could be explained in light of the crucial rules of ammonia and CTAB and the produced internal pressure within the autoclave reactor through such hydrothermal process in (i) dehydrogenation of small molecules, (ii) forming large planar aromatic macromolecules, followed by (iii) condensation polymerization, and finally (iv) reducing the O-content of the formed graphitic material. This is attributed to the evolved hydrogen gas within the autoclave during the hydrothermal process, in view of the fact that ammonia undergoes partial thermal decomposition, and the produced H_2_ gas effectively reduces the aromatic domains which constitute the graphitic structures. Besides, CTAB’s thermal decomposition rate reaches around 270 °C after hydrothermal treatment, resulting in many hydrogen-containing gases and significantly reducing the produced oxygenated graphitic structure^33–35^. The above results demonstrate that the obtained material is considered a highly graphitized condensed structure, formed through the dehydrogenation of small molecules, which can be promoted by the effect of thermal decomposition of CTAB and the pressure within the autoclave system, to form large planar aromatic macromolecules, followed by condensation polymerization.

#### Raman analysis

**Fig. 5:**
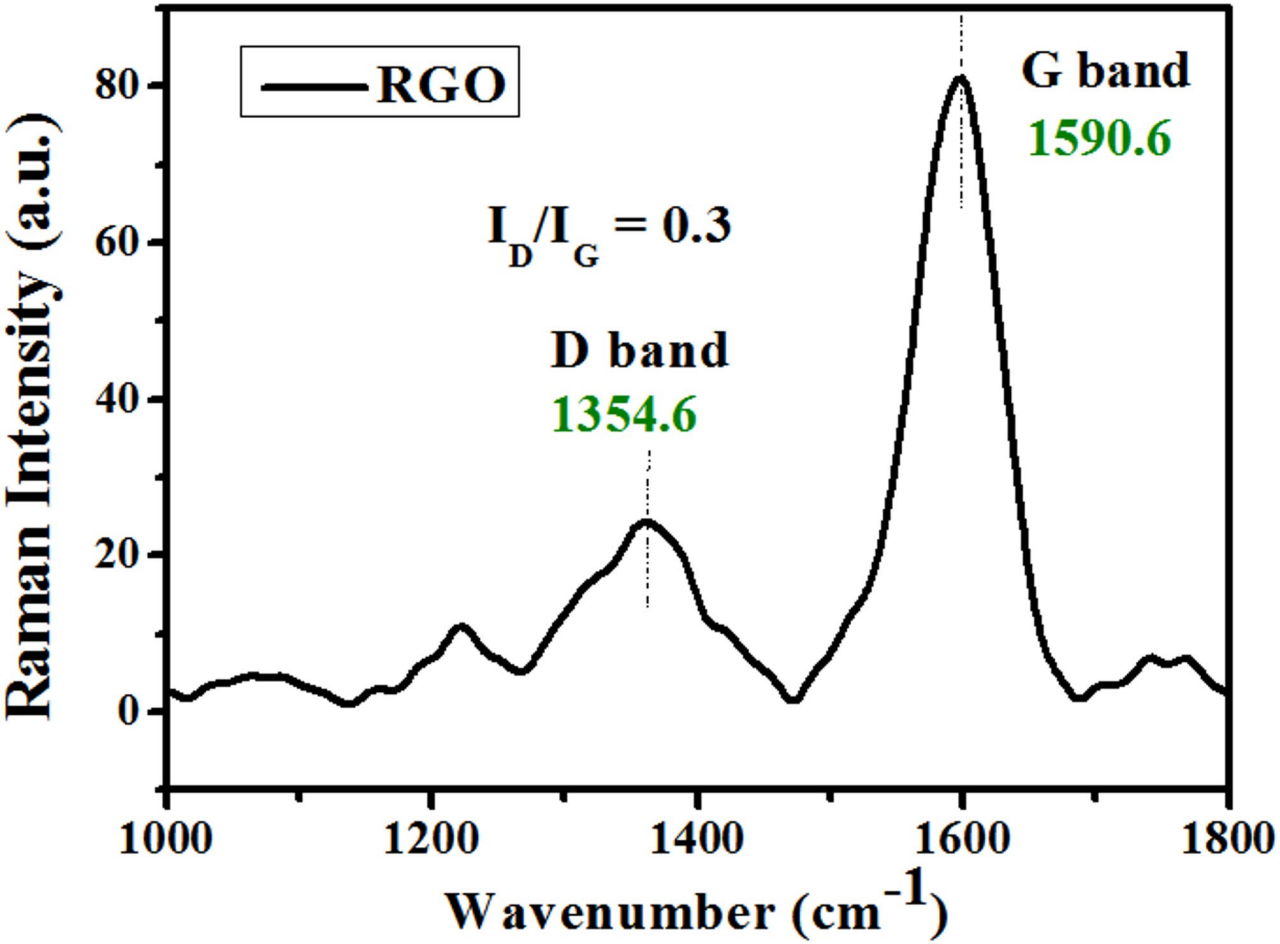
Raman spectrum of the as-prepared highly graphitized reduced graphene oxide HG-RGO.

The Raman spectrum of the as-prepared graphitic material HG-RGO is presented in Fig. 5. The spectrum reveals graphene’s two Raman characteristic bands, D and G, at 1354.6 and 1590.6 cm^− 1^, respectively. Those are due to graphitic lattice disorder and in-plane vibration of the aromatic sp^2^ carbon (C=C), respectively^35^. The peak intensity ratio (I_D_/I_G_) of HG-RGO is only 0.3, significantly lower than the values typically reported for other graphitic materials, indicating a higher degree of graphitization^8,9,36,37^.

Additionally, to further assess the structural order/disorder of the graphitic material, the lateral size of the aromatic sp^2^ domains (L_a_), which is expressed by the average in-plane crystallite size, was computed utilizing the Tuinstra and Koenig relation^33,34^, which is stated below Eq. 3

3 $${{\text{L}}_{\text{a}}}={\text{ C}}({\text{l}})/\left( {{{\text{I}}_{\text{D}}}/{{\text{I}}_{\text{G}}}} \right)$$

C(λ) is a wavelength-dependent constant, considered 4.4 nm ^33,34^. Based on this equation, the average in-plane crystallite size (Lₐ) was determined to be 14.7 nm. The combination of a low I_D_/I_G_ ratio and a large lateral crystallite size confirms the formation of a highly ordered graphitic structure. This structure likely results from the dehydrogenation of small molecules followed by condensation polymerization, leading to the growth of large, planar aromatic macromolecules.

#### HR-TEM and SAED pattern

**Fig. 6:**
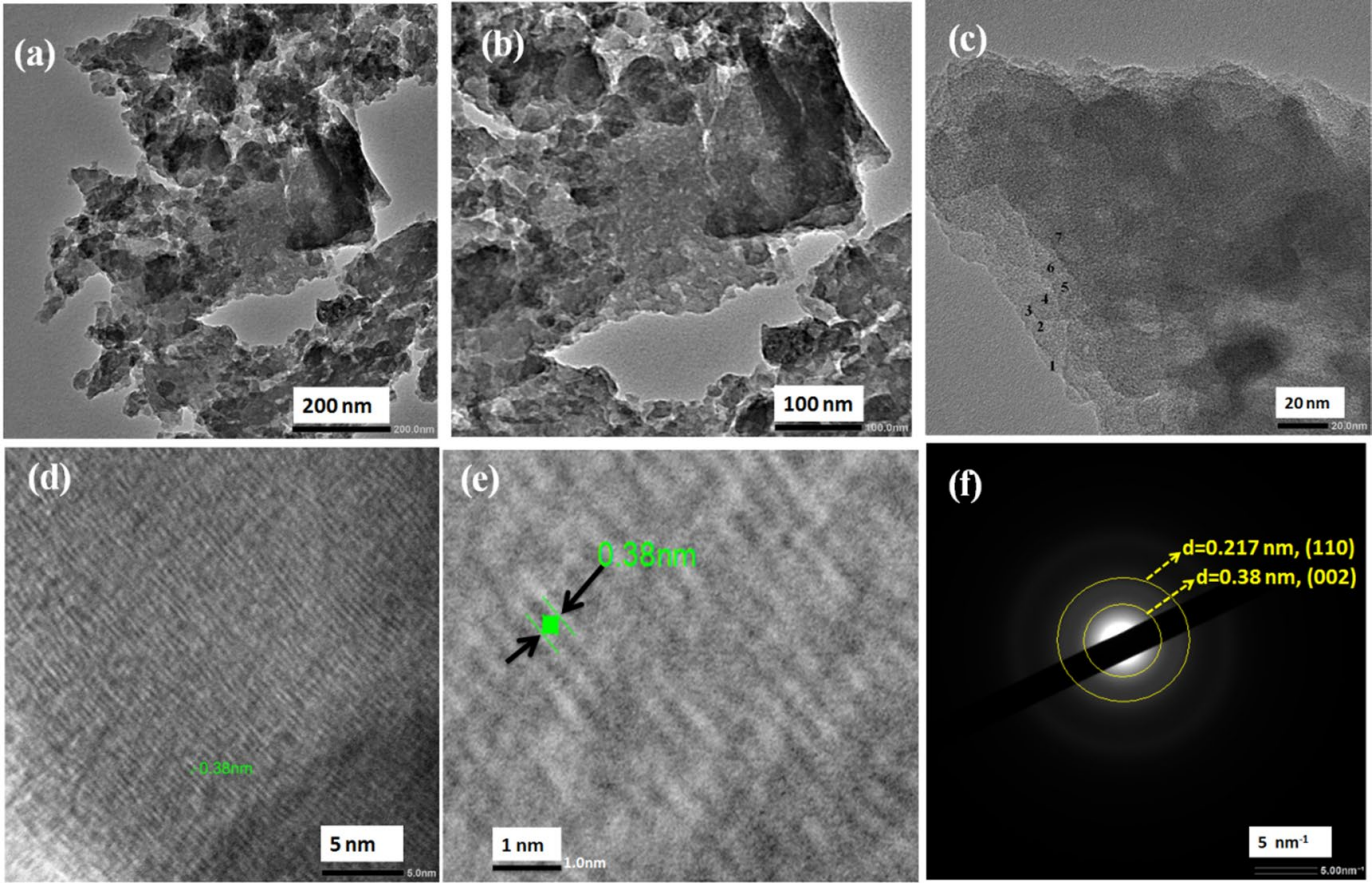
(**a**, **b** and **c**) TEM images of the as-prepared highly graphitized reduced graphene oxide HG-RGO, (**d**, **e**) HR-TEM images and (**f**) SAED patterns.

Figure 6 shows the TEM images of the as-prepared highly graphitized reduced graphene oxide. The sheet morphology has been verified from the TEM images, Fig. 6(a, b and c), with an extended lateral dimension of about 0.5 μm. The observed sheets have varying thickness, which is evidenced by the darkness in specific parts and lightness in others. These sheets are magnificent and resemble well-separated flat morphology with folding and wrinkling features, Fig. 6(a, b). These sheets have approximately seven layers, as seen in Fig. 6(c). Figure 6(c, d) estimated the interplanar distance from the high magnification images. It was found to be ≈ 0.38 nm, corresponding to the lattice plane (002) of RGO, which is in good agreement with that of RGO^38^. Figures (6f)implies the SAED patterns of the HG-RGO with a scale bar of 5 nm^− 1^. The lattice-fringe spacing recorded in HRTEM micrographs was measured utilizing digital image analysis of reciprocal space distinctive features. The analysis used the Digital Micrograph program (a Java-based graphic design program, Image- software). The acquired Lattice fringes also confirm that the interplanar distance matches for RGO material, which is 0.38 and 2.17 nm, corresponding to the lattice planes (002) and (110), respectively. It was confirmed that the main phase is RGO, consistent with the synthesised graphitic material’s XRD results. It shows the lattice fringes with a spacing of 2.13 Å corresponding to the carbon (110) plane^33^.

HR-TEM imaging discloses RGO nanosheets with stacked atomic carbon layers up to ~ 7 layers locally in certain regions; however, Scherrer’s equation in XRD analysis gives an average coherent sacking (crystallite thickness) corresponding to ~ 4–5 layers across the entire sample. The as-prepared HG-RGO is evaluated as a multilayer graphitic material (the number of stacked atomic layers does not exceed ten).

A modest yield of HG-RGO is obtained (40 wt% ), and the product possesses good structural characteristics as revealed by all the presented analysis findings. Regarding reproducibility, the yield varies by less than 3% between two runs, demonstrating good reproducibility. Concerning the scalability, a Teflon-lined autoclave is simply a sealed reactor. Hence, the hydrothermal procedure can be straightforwardly scaled up by enlarging the volume of the sealed reactor, which is certainly accessible in industry.

That combination of these features that characterize such HG-RGO synthesis route, simplistic, cost-effective, scalable, eco-friendly, good yield and excellent quality product, validates that it can be valuable for the industrial production of HG-RGO. Thus, owing to a cheap feedstock and lessened energy demand, we hold a promising trend for lower production cost than conventional GO or CVD-based methods.

### The comparative analysis of various synthesis strategies for coal Tar pitch (CTP)-derived graphitic materials

The comparative analysis of various synthesis strategies for coal tar pitch (CTP)-derived graphitic materials is presented in Table 3. It highlights the diversity in methodological approaches and the significant influence of processing conditions on the final material properties. CTP, a by-product of coal tar distillation, offers high aromatic content and low cost, making it an attractive carbon precursor for graphitic nanostructures. However, its transformation into highly graphitized forms requires careful optimization of synthesis parameters. Several studies have employed different techniques such as hydrothermal treatment, chemical vapour deposition (CVD), template-assisted growth, and microwave heating to enhance the graphitization process. For instance, researchers have introduced carbon nanotubes (CNTs), carbon black (CB), silicon carbide (SiC), and metallic catalysts (e.g., Fe, Ni) as additives to promote the formation of ordered graphitic structures. These additives often serve multiple purposes, as nucleating agents and structural templates. Using soft or hard templates (e.g., SiO₂, MgO, FeOₓ) helps configure the carbon structure during pyrolysis, leading to the formation of graphene-like sheets or carbon nanosheets (CNSs). However, these methods often require post-synthesis template removal steps, such as acid washing, which increase the process’s complexity, cost, and environmental footprint. Here, CTAB was proposed not as a hard or constant structural template, but somewhat as a dispersing agent and temporary surfactant that facilitates the self-assembly of graphitic domains and exfoliation process during hydrothermal treatment. Processing temperature and atmosphere are also critical factors. High-temperature treatments (> 1000 °C) under inert gases like argon (Ar) or reducing environments facilitate aromatization and promote the formation of sp^2^-hybridized carbon networks. However, such high temperatures may not be energy-efficient for large-scale industrial applications. This limitation has led to growing interest in low-temperature synthesis routes or template-free methods, which aim to simplify processing while achieving desirable graphitic characteristics.

Interestingly, some recent approaches explore eco-friendly solvents and biomass-based additives, aligning with the broader goal of sustainable material synthesis. For example, hydrothermal synthesis in the presence of surfactants like cetyltrimethylammonium bromide (CTAB) offers a low-cost and relatively low-temperature route for developing nitrogen-doped reduced graphene oxide (N-RGO) or highly graphitized carbon materials. Overall, this comparative study underscores the balance between performance and practicality. While many methods produce high-quality graphitic materials, challenges remain regarding scalability, energy consumption, and environmental impact. Thus, ongoing research is expected to refine these strategies, possibly through hybrid techniques or the integration of green chemistry principles to achieve industrially viable, high-performance graphitic materials from coal tar pitch.

**Table 3:** The comparative analysis of various synthesis strategies for coal Tar pitch (CTP)-derived graphitic materials.

Method	T (°C)	Additives	The produced graphitic material	Atmosphere	Washing solvent	Used Template	I_D_/I_G_	References
Heat treatment of CTP and acid-treated CNT and CB	1000 2000	CNT &CB	Graphitized carbon residue	high-purity Ar atmosphere	–	–	0.68	^6^
CTP-based carbons modified with nSiC were	– 1000 1000 2000 2800	SiC Nanoparticles	CTP-derived carbon residue CTP-pure CTP-10%SiC-1000 CTP-10%SiC-2000 CTP-10%SiC-2800	Ar atmosphere	ethanol	–	3.8 5.7 0.35 0.35	^7^
Chemical vapour deposition processes	200 for 30 min 800 for one hour	–	Graphene capsules	N_2_ atmosphere	2 M HCl&distilled water	nano-MgO& KOH	A strong D-band indicates that the as-made HPGBs have a low graphitization degree	^8^
Activation by conventional and microwave heating (output power of 600 W)	30 min.	–	Hierarchical porous carbons (HPCs)	N_2_ atmosphere	Conc. H_2_SO_4_	Nano-sized ɤ-Fe_2_O_3_ as a template and KOH	--	^39^
layered-template-nanospace-confinement strategy coupled with in-situ chemical activation	200 for 30 min 800 for 1 h	–	corrugated graphenenanosheets	Ar atmosphere	2 M HCl solution and distilled water repeatedly	MgO templates& KOH	0.84	^9^
Hydrothermal	270	–	HG-RGO	Air	Distilled H_2_O	Hard template -free	0.3	**Current study**

### Characteristics of GMB for asphalt mixture

#### Physical characteristic of aggregates

Aggregates were sieved, starting with coarse particles retained on the size of the sieve 1” and moving to fine particles retained on a sieve width of 0.075 mm (No. 200) (filler). All particles were graded following the Egyptian Code’s acceptable gradation limitations^40^. The asphalt also contained two types of coarse dolomite, grade (I) and grade (II), obtained from the “ATAKA” quarry in Suez, Egypt, with the physical parameters reported in Table 4. Sufficient silica sands, obtained from the “ELREHAB” quarry in Cairo, Egypt, having an average specific gravity of 2.62 g/cm3, as well as limestone dust having a specific gravity of 2.64 g/cm3 (filler), were utilized as asphalt concrete materials, as illustrated in Table 5. Three tests for aggregate characterization were applied: Los Angeles abrasion, water absorption, and specific gravity. The percentage of flat particles, elongated particles, clay lumps, friable particles, and fractured particles was also estimated. In addition, the liquid and plastic limits for aggregates were estimated.

**Table 4:** Physical properties of coarse and fine aggregates.

Properties	Aggregate Gradation AASHTO T -27		
	Coarse	Fine	Standard
Bulk specific gravity (g/cm^3^)	2.724	2.571	AASHT T-85
Specific gravity-saturated dry surface (g/cm^3^)	2.749	2.723	AASHT T-85
Apparent specific gravity (g/cm^3^)	2.795	2.794	AASHTO T-85
Absorption (%)	0.9	1.5	AASHTO T-85
Abrasion test after 100 cycles (%) After 500 cycles (%)	4 19	4 19	AASHTO T-96
Flat particles (%) Elongated particles (%)	1 0	1 1	ASTM D-4791
Clay Lumps &friable particles (%)	0.1	0.2	AASHTO T-112
Fractured particles	2	3	ASTM D-5821
Plastic limit	0	0	AASHTO T-90
Liquid limit	0	0	AASHTO T-89

**Table 5:** Properties of the used filler.

Type of filler		Limestone dust	
Bulk specific gravity (g/cm^3^)		2.627	AASHT T-85
Specific gravity-saturated dry surface (g/cm^3^)		2.648	AASHT T-85
Apparent specific gravity (g/cm^3^)		2.684	AASHT T-85
Absorption (%)		0.8	AASHT T-85
Sieve analysis			AASHTO T-27
Sieve #	% Passing		Specs
No.50	100		95–100
No. 100	91		85–95
No.200	66		65–85

#### Job mix formula of aggregates

**Fig. 7:**
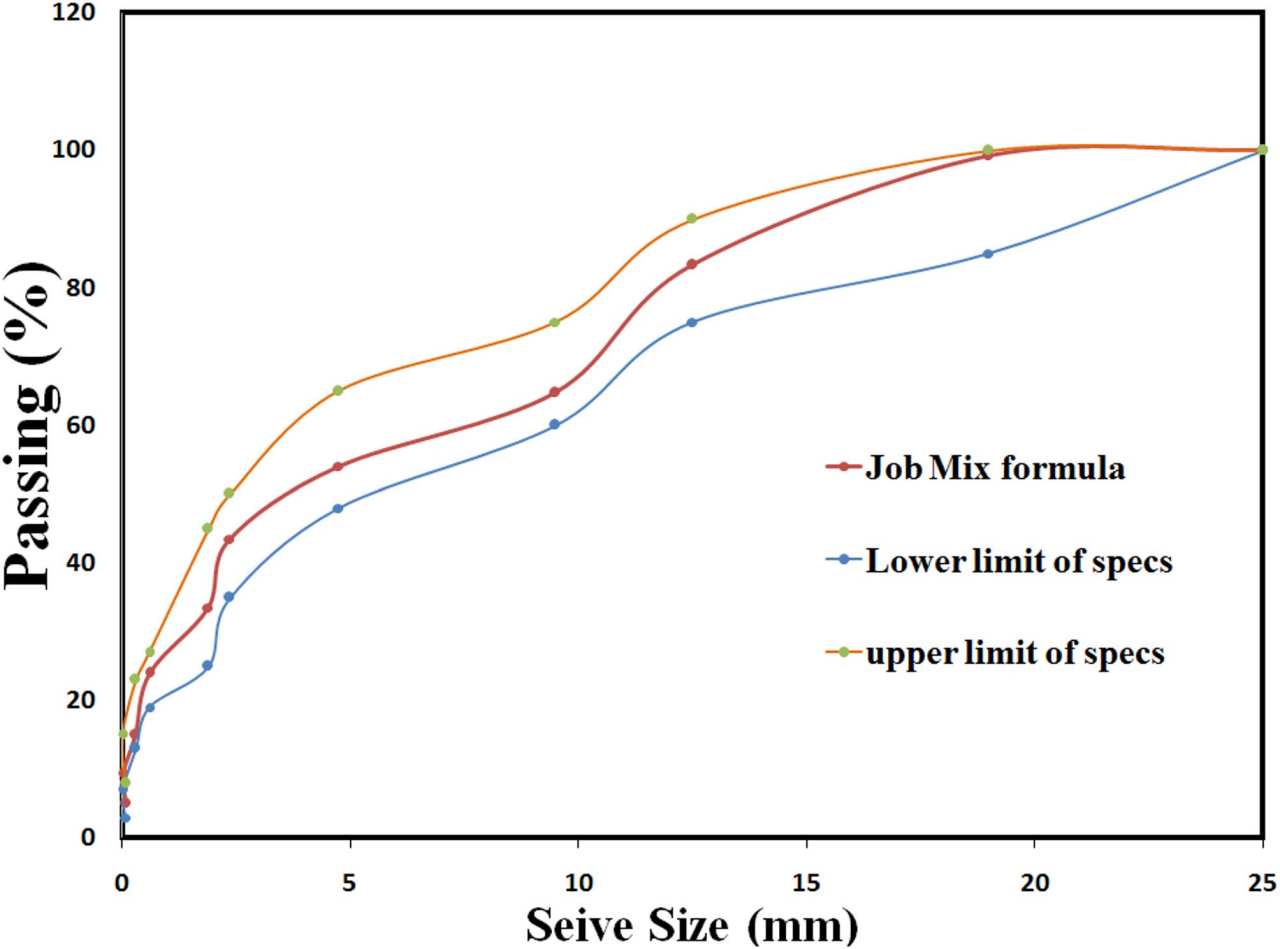
Job Mix Formula (JMF) of solid materials.

The aggregate gradation, as illustrated in the provided Fig. 7, is a fundamental property that dictates the performance characteristics of an asphalt mixture, including its workability, compatibility, strength, and durability. This graph effectively presents the particle size distribution of the proposed Job Mix Formula (JMF) compared to the established Lower and Upper Limits of Specifications^41^.

Upon careful examination, it is evident that the Job Mix Formula curve (red line) falls entirely within the specified gradation envelope, defined by the lower limit (blue line) and the upper limit (orange line) across the full range of sieve sizes (0 to 25 mm). This precise fit indicates that the selected aggregate blend adheres strictly to the project’s or standard’s requirements for particle size distribution^42^.

### Effect of HG-RGO on the physical properties of bitumen

To thoroughly examine the impact of Highly-Graphitized Reduced Graphene Oxide (HG-RGO) on the physical properties of bitumen, a series of conventional asphalt binder tests was conducted, with key findings summarized in Table 6. A prominent observation was the significant hardening effect on bitumen 80/100 upon adding HG-RGO. Specifically, the penetration value notably decreased to 45, representing an approximate 52.9% reduction compared to the control sample’s 85 dmm. Concurrently, the softening point temperature remarkably increased to 60 °C, indicating a 75% enhancement relative to the unmodified binder. This pronounced improvement in both penetration and softening point is likely attributable to the formation of an exfoliated or well-intercalated HG-RGO structure within the bitumen matrix, where the graphene sheets provide strong physical reinforcement, thereby imparting high-temperature performance and resistance to deformation. Consistent with this hardening trend, the kinematic viscosity of the bitumen was observed to increase directly with rising HG-RGO content, a finding corroborated by numerous studies in the literature^43–45^, which further underscores graphene’s ability to stiffen the binder’s consistency. However, it was critically noted that while low to moderate percentages of HG-RGO enhance high-temperature performance, a high percentage (specifically 5 wt%) negatively affected the elasticity of the bitumen. This suggests that excessive loading of HG-RGO can lead to an overly stiff or brittle binder, potentially compromising its low-temperature performance or fatigue resistance, thus advising against excessively high HG-RGO concentrations for optimal bitumen modification^46^.

**Table 6:** Physical properties of bitumen 80/100 and GMB.

Physical properties	Bitumen 80/100	GMB1 1wt% HG-RGO	GMB3 3wt% HG-RGO	GMB5 5wt% HG-RGO	Standard
Penetration(@25°C, 100 g,5s) 0.1 mm	85	75	68	45	ASTM D5
Softening point (ring and ball) °C	45	51	55	60	ASTM D36
Ductility @ 25 °C, (5 cm/m) cm	100	100	100	84	ASTM D113
Kinematic viscosity (@ 135 °C) cSt.	300	435	550	620	ASTM D2170
Tests after Ageing by RTFOT at 163 °C for 6 h (ASTM D2872)					
Penetration (@ 25 °C, 100 g,5s) 0.1 mm (% from original value)	60	45	40	38	
Ductility 25 °C (5 cm/m) cm %	50	50	50	50	

### Effect of HG-RGO on the dynamic mechanical properties of bitumen binder

Characterizing the rheological properties of bitumen binders is crucial for accurately predicting major pavement failures like rutting, ravelling, and stripping. To examine the viscoelastic properties of the GMB (Graphene Modified Bitumen), a laboratory study was conducted using a Dynamic Mechanical Analyzer (DMA). This involved performing two sweep tests on all bitumen binder samples to thoroughly investigate the effects of both temperature and time on the performance of the virgin and modified binders.

#### Temperature sweep

**Fig. 8:**
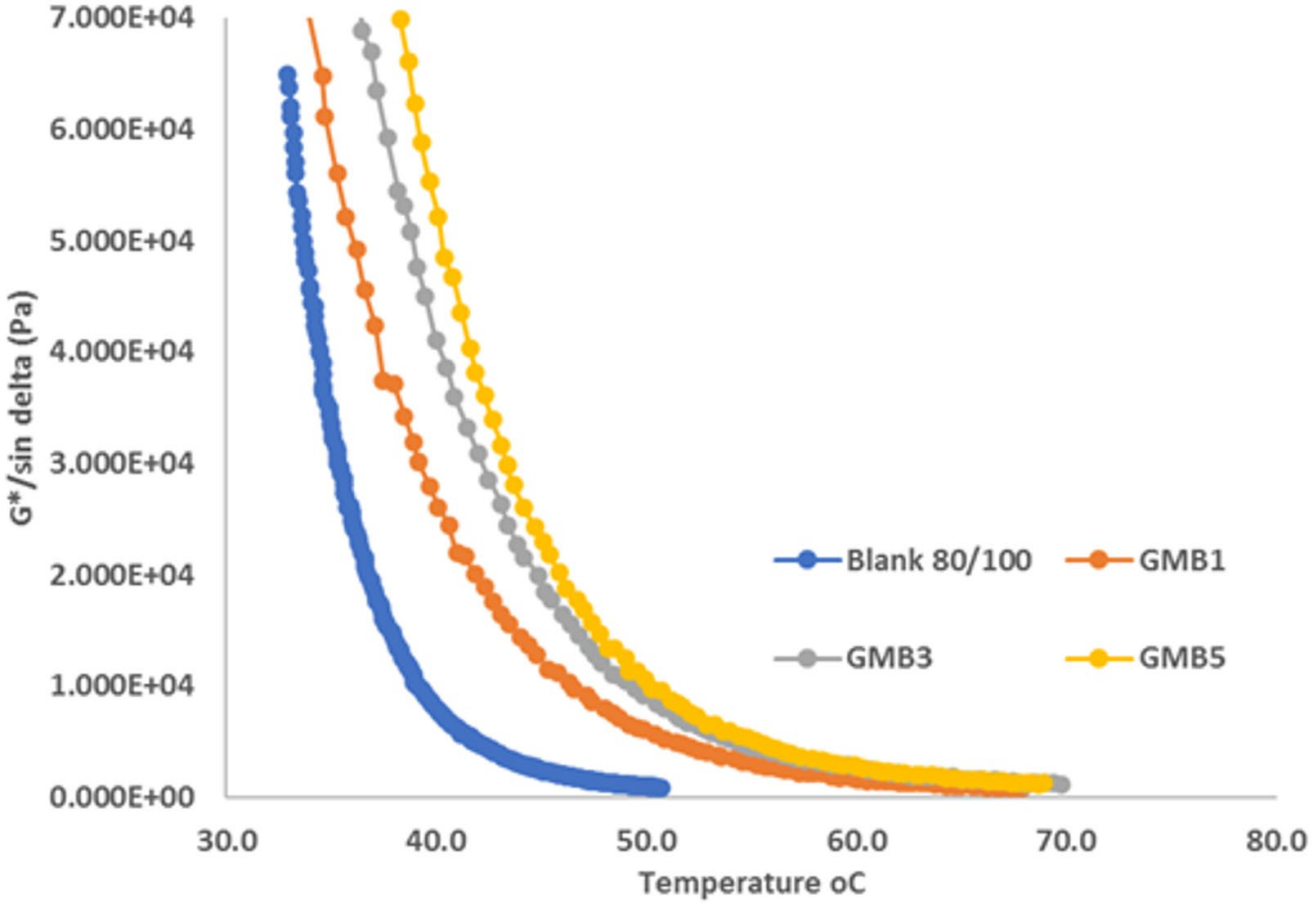
Effect of HG-RGO loading on rutting factor (G*/sindelta) of bitumen binder.

**Fig. 9:**
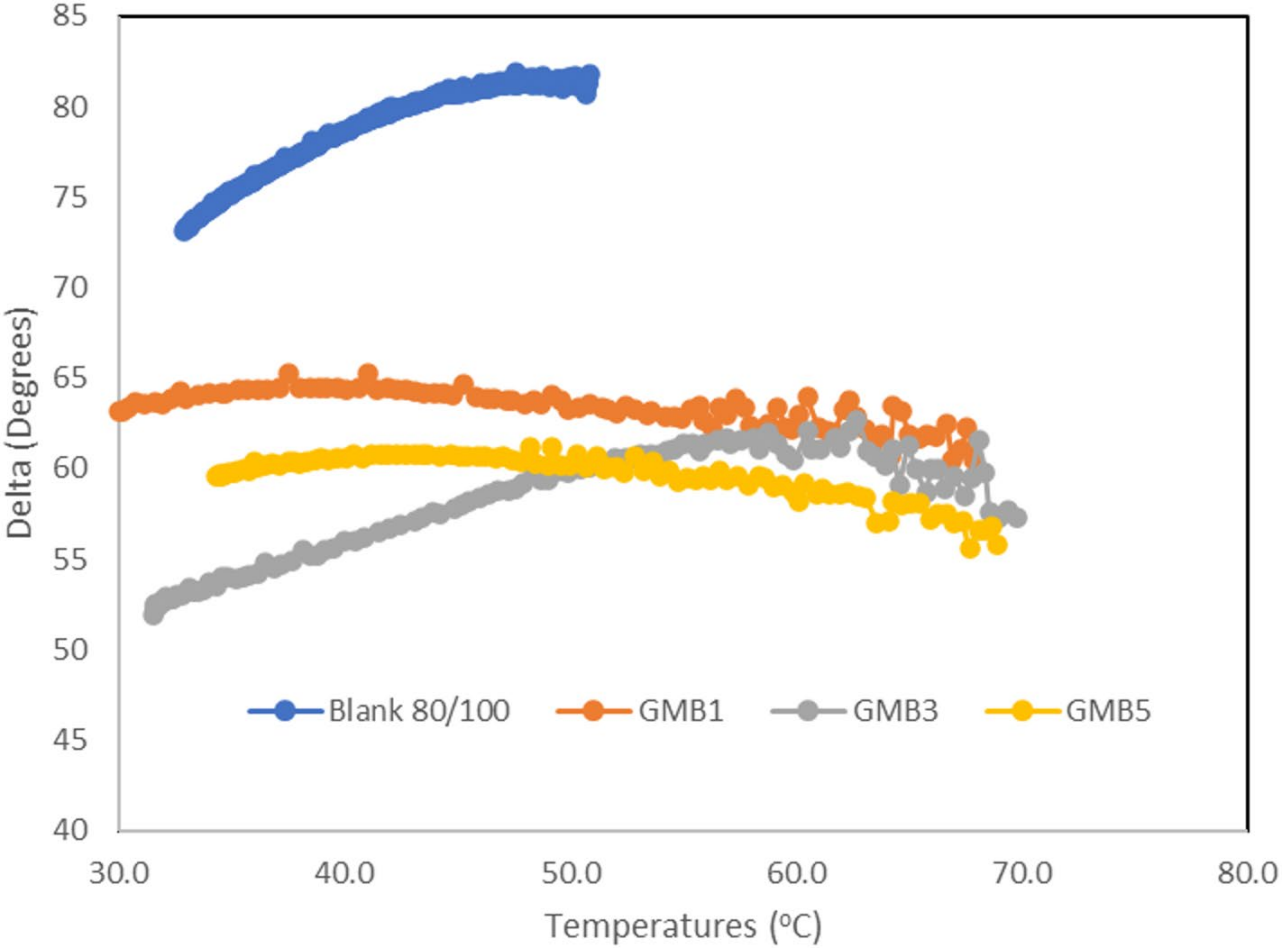
Effect of HG-RGO loading on phase angle (δ) of bitumen binder.

The dynamic mechanical analysis (DMA) results, comprehensively presented in Figs. 8 and 9, clearly elucidate the enhanced viscoelastic performance conferred by the Highly-Graphitized Reduced Graphene Oxide (HG-RGO) modifier. As anticipated for viscoelastic materials of bitumen, Fig. 8 demonstrates that an increase in temperature leads to a corresponding decrease in the complex modulus (G^∗^) and, critically, the rutting resistance parameter (G^∗^/sin(δ)) ^47^. This is fundamentally rooted in the thermal behaviour of asphaltenes: at lower temperatures, asphaltenes form a stiff, compact internal structure, while at higher temperatures, thermal energy overcomes intermolecular forces, causing the asphaltenes to disperse into individual particles. Consequently, the bitumen binder transitions into a liquid-like state, and the phase angle (δ), the time lag between applied stress and resultant strain, increases dramatically with temperature. Also, it was noticed that the addition of HG-RGO in Fig. 8 demonstrates that all HG-RGO modified bitumens exhibit a significantly greater G^∗^/sin(δ) value than the base bitumen at the same temperature. The base bitumen’s rutting factor falls below the AASHTO standard limit of 1.0 kPa at 50 °C, indicating poor resistance to permanent deformation. In stark contrast, all HG-RGO modified bitumen maintains a rutting factor above this threshold at 50 °C, unequivocally confirming their high rutting resistance. This improvement stems from the nano-scale effects of HG-RGO: its large active surface area, high surface energy, and quantum effects facilitate strong physical binding with the asphalt matrix and promote a good, uniform dispersion within the base bitumen (iv).

The changes in the phase angle (δ) (Fig. 9) further confirm the modification’s impact on elasticity. For the base asphalt, the phase angle steadily increases from 75 °C to 85 °C as the temperature rises from 35 °C to 55 °C, indicating a rapid shift toward viscous (liquid) behaviour. Conversely, the HG-RGO modified asphalts (GMB1, GMB3, and GMB5) exhibit lower phase angles than the base bitumen at the same temperatures. This reduction in the phase angle signifies an increase in the elastic component of the binder, reinforcing the bitumen’s structure and improving its ability to recover from strain, which is a direct mechanism for enhanced rutting resistance^48^. The differing thermal trends in phase angle between modified and base bitumen underscore that the incorporated HG-RGO nanoparticles physically inhibit the free movement and dispersion of the asphaltene matrix at elevated temperatures, thus stabilizing the binder’s elastic component^49^.

#### Fatigue parameter

**Fig. 10:**
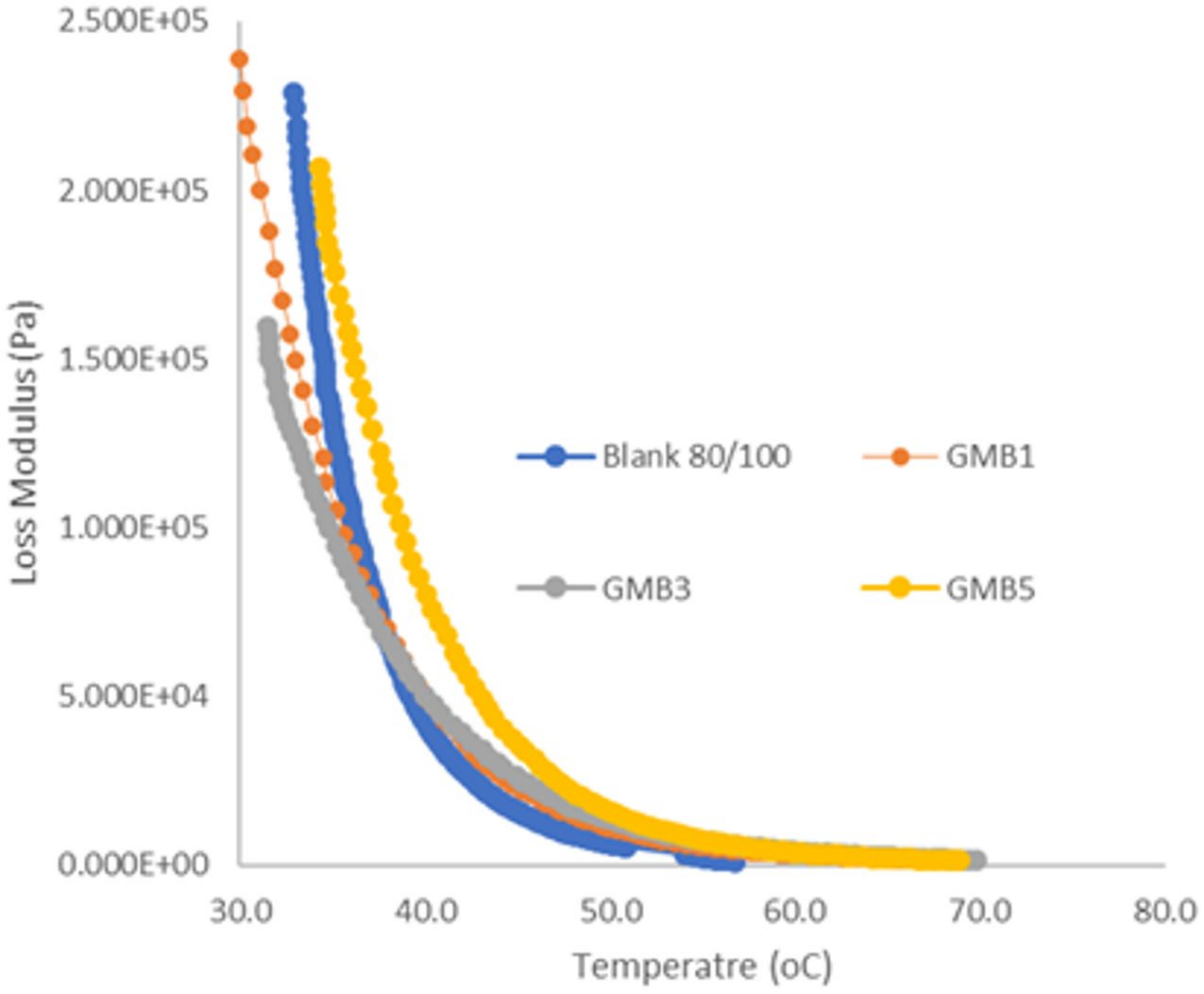
Effect of HG-RGO on fatigue parameter for reduced graphene modified bitumen.

**Fig. 11:**
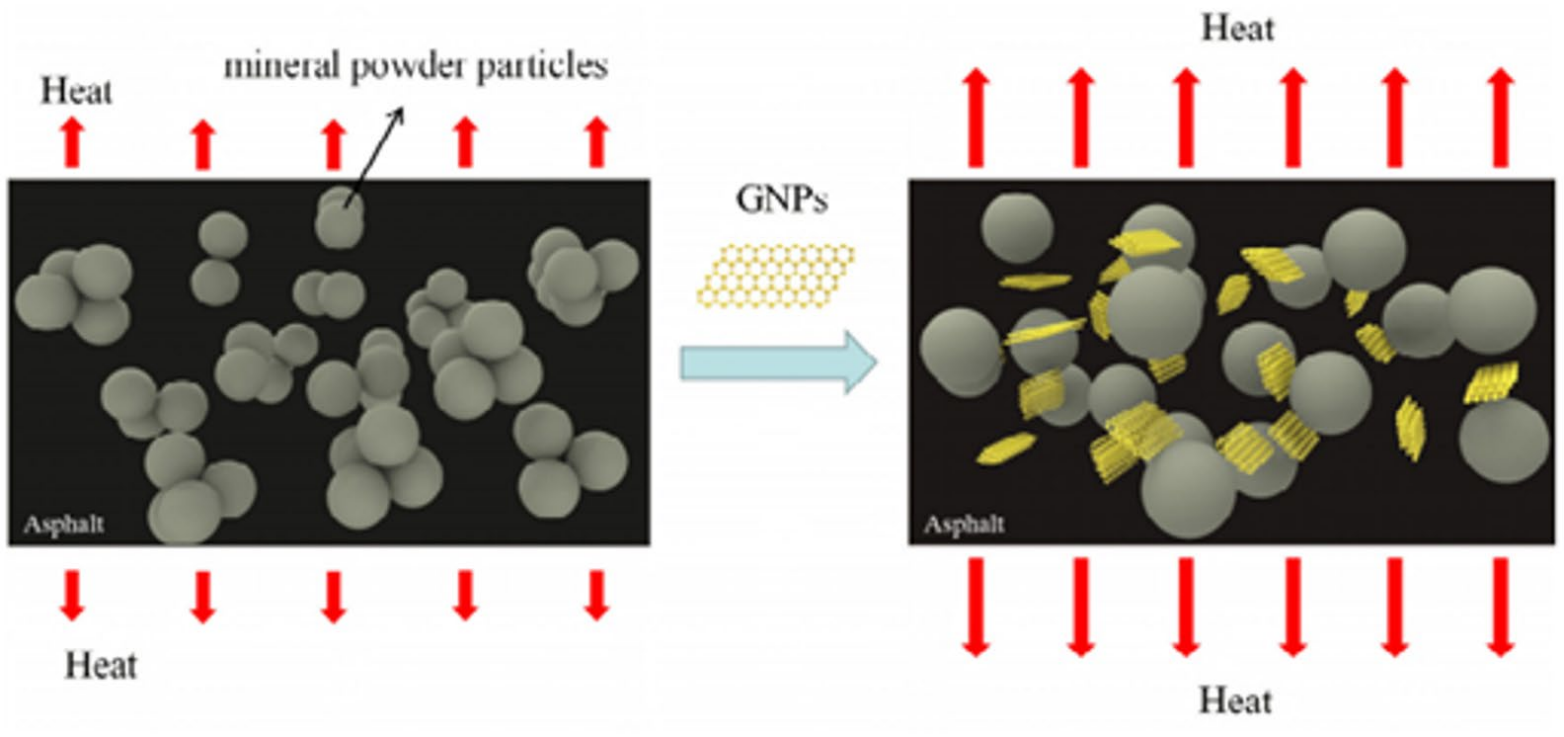
Mechanism of loading graphene to bitumen depicted from^49^.

Fatigue cracking represents a critical failure mode in asphalt pavements, fundamentally linked to the magnitude of dissipated energy generated under repeated external loading. The loss modulus (E*sinδ) is universally adopted as a key rheological parameter to quantify the binder’s resistance to fatigue, where a lower value corresponds to superior fatigue resistance. This study evaluated the fatigue parameter for all bitumen samples at a standard temperature of 35 °C and a frequency of 1.59 Hz. The data (as illustrated in Fig. 10) clearly indicate that the incorporation of Highly-Graphitized Reduced Graphene Oxide (HG-RGO) significantly reduced the fatigue parameter, suggesting the Graphene-Modified Bitumen (GMB) binder is capable of dissipating less energy under cyclic loading. Specifically, the base bitumen registered a G^∗^sinδ of 132 kPa, which was substantially lowered to 113 kPa for GMB1 (1wt %) and 110 kPa for GMB3 (3wt %), confirming enhanced fatigue life. The greater fatigue resistance offered by graphene modification stems from a multi-faceted synergistic mechanism surpassing traditional nanofillers^49^. Firstly, unlike simple mineral particles encapsulated in asphalt, graphene is interwoven with the bitumen matrix, creating an interlayer adsorption effect that significantly strengthens the bitumen’s cohesion and toughness, thereby increasing its complex modulus^50^. This unique structure allows the graphene sheets to distract and buffer a portion of the external shear stress, effectively preventing the initiation and propagation of micro-cracks^51^. Secondly, the excellent lubricating performance of graphene comes into play during cyclic shearing; the lubricating effect between the bitumen components and graphene sheets alters particle positions, reducing internal friction and stress concentration^52^. Finally, graphene’s super thermal conductivity plays a role by efficiently transferring and dissipating the heat generated by friction under loading, thus preventing localized heat concentration that often accelerates asphalt damage^19,53^. The mechanism was shown in Fig. 11. However, this study noted that adding 5wt% HG-RGO slightly increased the fatigue parameter to 125 kPa (for GMB5), suggesting a potential trade-off where high agglomeration at higher filler content diminishes the synergistic benefit^48^. Consequently, the optimal HG-RGO content for maximizing fatigue resistance was identified as 3wt%.

#### Time sweep test

**Fig. 12:**
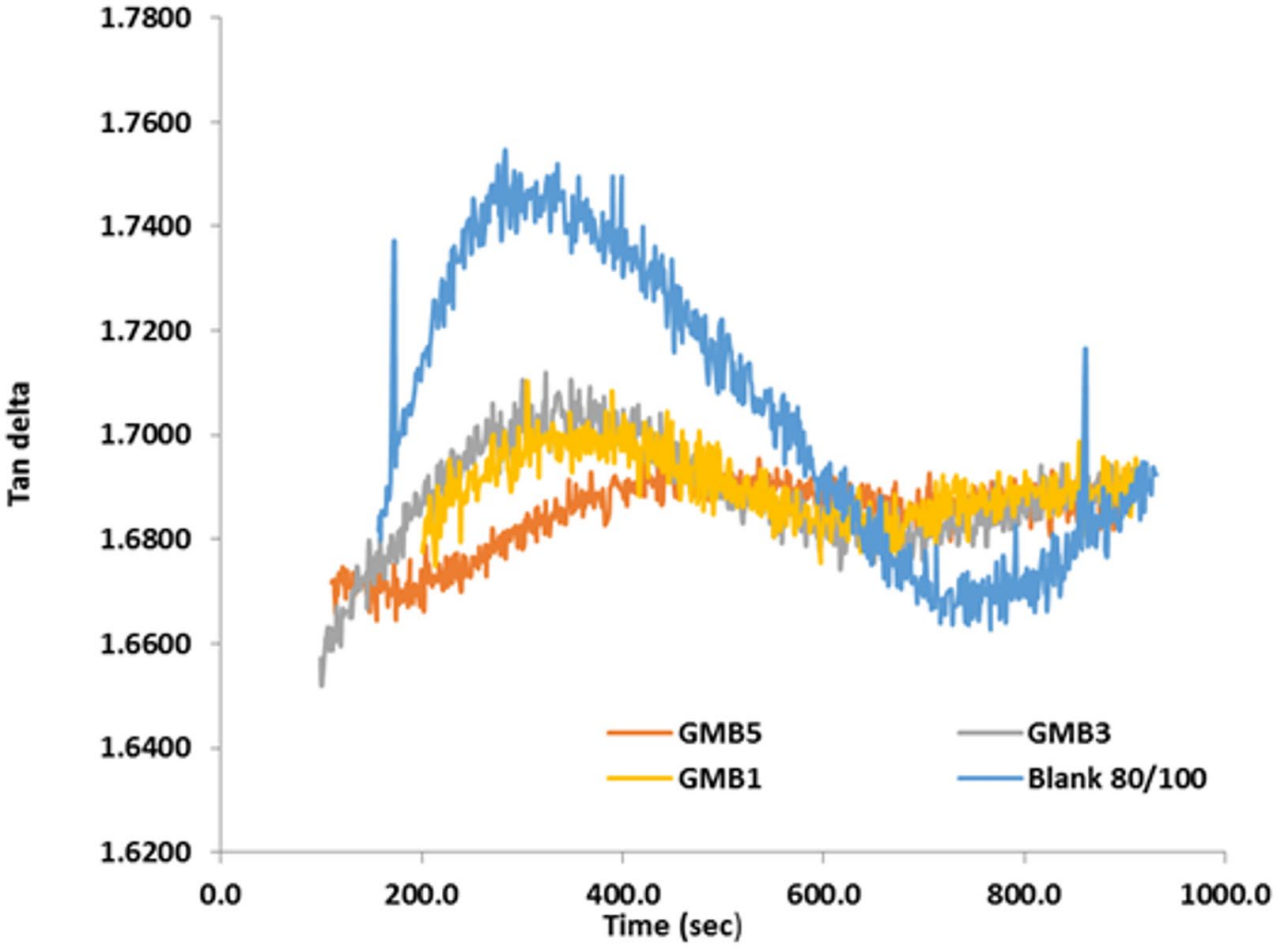
Effect of time on complex modulus for base binder and GMB.

**Fig. 13:**
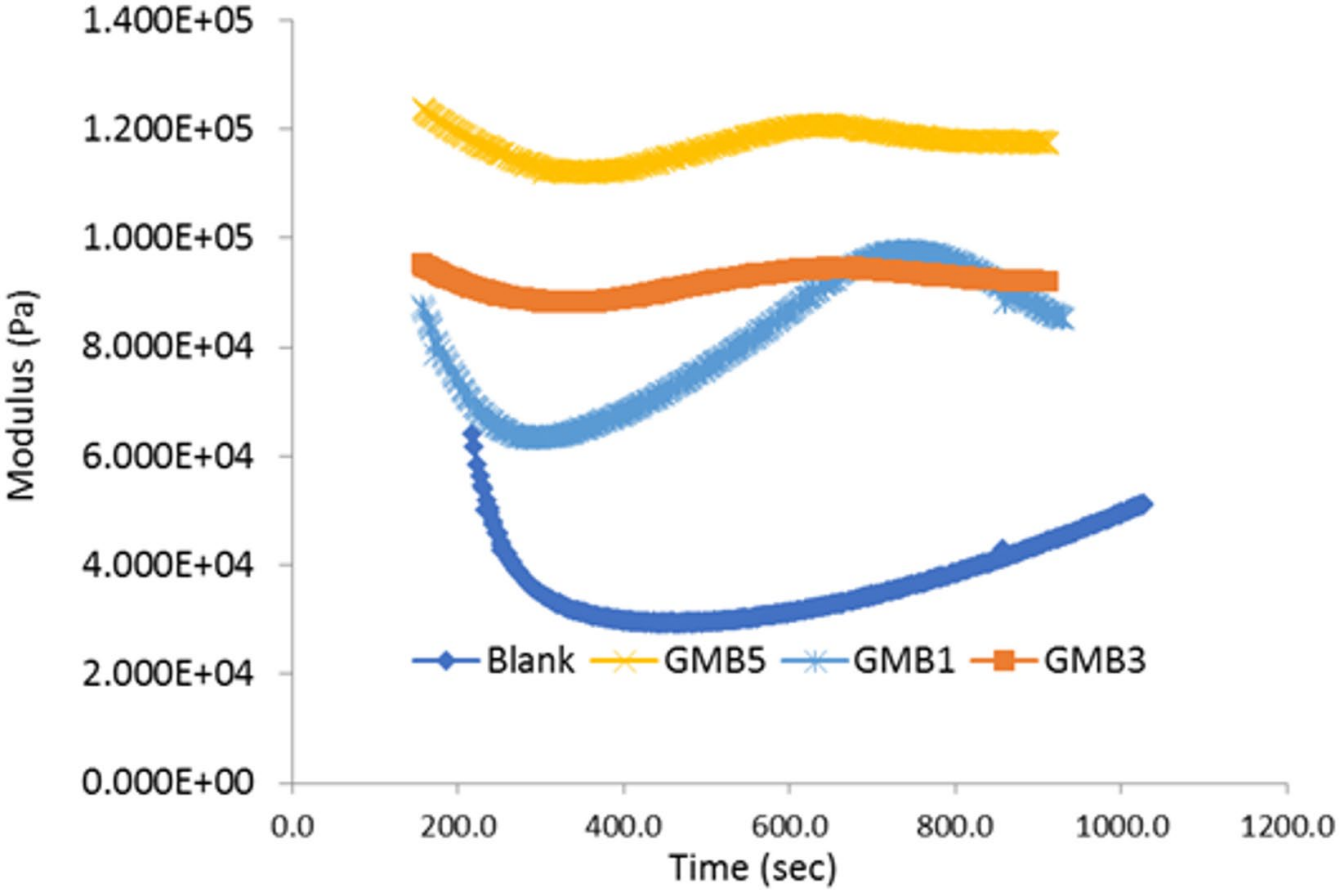
Effect of time on loss tangent of base binder and GMB.

The time-dependent viscoelastic response of the base bitumen and graphene-modified binders (GMB) was meticulously evaluated at 35 °C using time sweep tests, as illustrated by the complex modulus (G^∗^) and phase angle (tanδ) data in the respective figures (Figs. 12 and 13). The graphene-modified binders, specifically GMB3 and GMB5, exhibited remarkable rheological stability, showing only a slight decrease in G^∗^ and virtually no change in tanδ over the entire 15-minute testing period. This consistency is highly significant because, with constant strain and frequency, a stable viscoelastic response indicates a binder that resists structural changes under sustained mechanical stress. In stark contrast, the base bitumen displayed a sharp, noticeable decrease in both G∗ and tanδ over the same duration, signalling a considerable reduction in dynamic viscosity. This decline represents an apparent thixotropic softening effect, where the internal structure of the unmodified bitumen breaks down over time under constant stress^54^. For Hot Mix Asphalt (HMA) pavements, this implies that base bitumen is susceptible to softening even under minimal traffic or static conditions early in its service life, potentially leading to instability^55^. Conversely, the high stability demonstrated by the GMB binders confirms that the incorporation of graphene significantly reinforces the bitumen’s internal network, mitigating the thixotropic softening effect and resulting in a longer, more reliable service life for the pavement structure.

### Effect of HG-RGO on the mechanical properties of asphalt mixtures

The longevity and resilience of bituminous pavements under the relentless assault of traffic loading and diverse environmental conditions are primarily predicted through critical tests applied to the asphalt mixtures, notably Marshall stability, flow, and rutting resistance evaluations^56^. While the body of literature explicitly addresses asphalt mixtures prepared with graphene-modified bitumen, it is still emerging compared to traditional modifiers, and many studies consistently report promising results ^57,58^. These investigations, particularly those employing the Marshall stability and flow test, uniformly demonstrate that asphalt mixtures incorporating graphene exhibit significantly higher stability values and concurrently lower flow values than control mixtures prepared with bitumen pen grade 80/100. This consistent trend highly indicates graphene’s ability to fundamentally stiffen the bitumen matrix and enhance the internal friction and cohesion within the asphalt mixture^59^. Mechanistically, increased Marshall stability signifies a greater load-bearing capacity and resistance to plastic deformation, while reduced flow values suggest that the mixture can withstand deformation without excessive yielding. These improvements directly translate to high rutting resistance in the field, implying that pavements constructed with graphene-modified bitumen are poised to offer enhanced durability and a longer service life under demanding traffic conditions^60^.

#### The design of Marshall for asphalt mixture

**Fig. 14:**
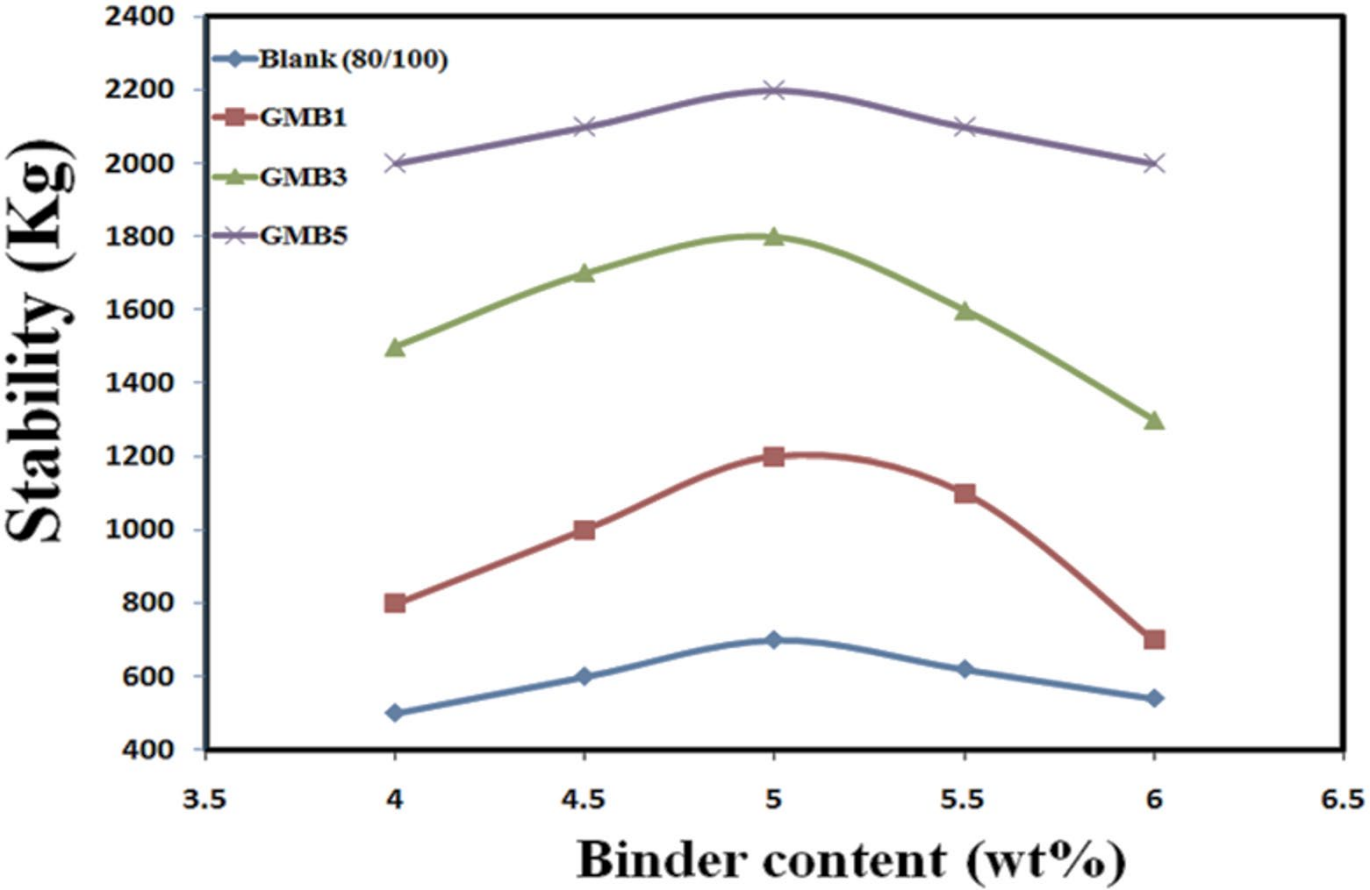
Marshall Stability curves of GMB binders.

**Fig. 15:**
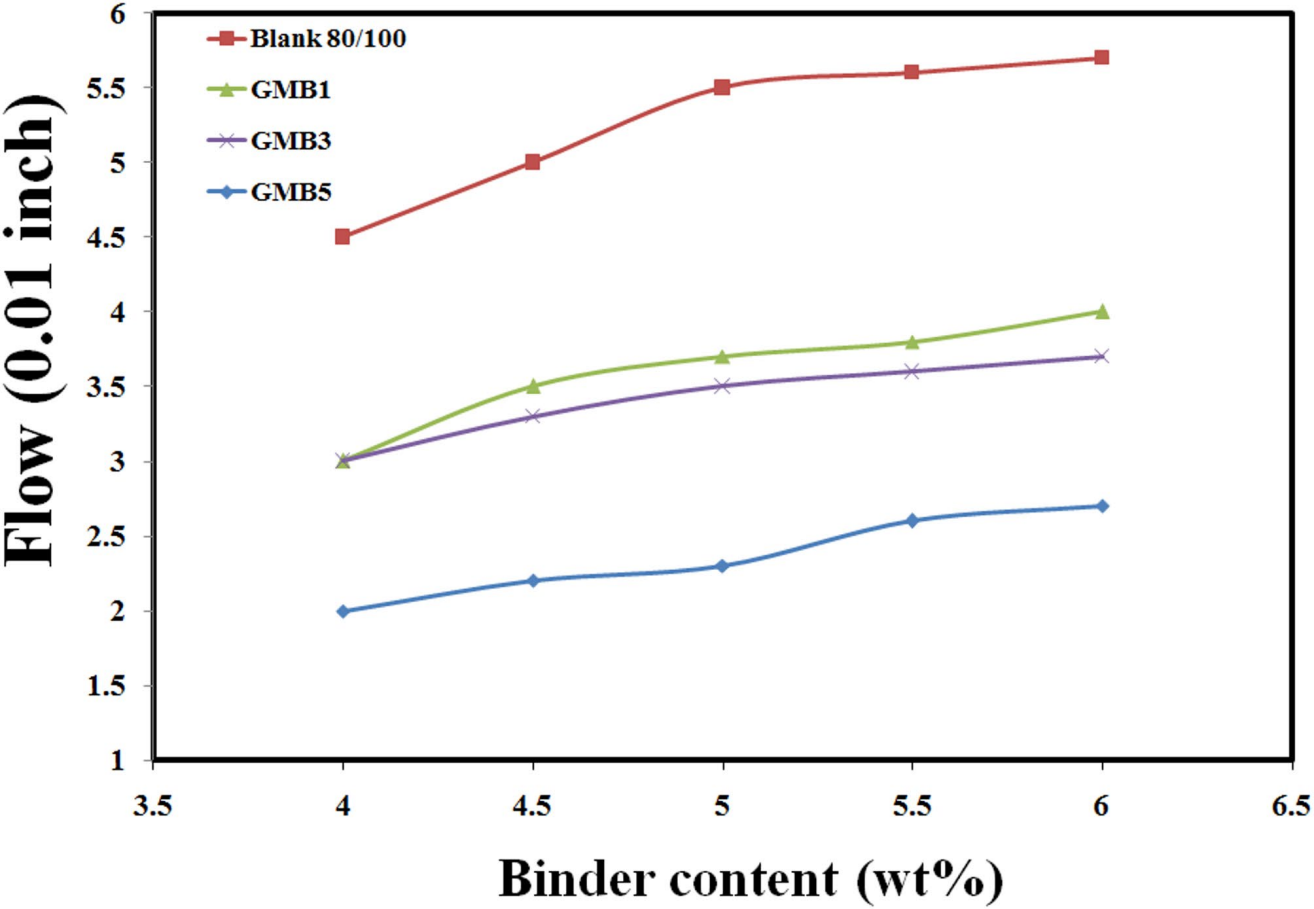
Flow of GMB binders.

**Fig. 16:**
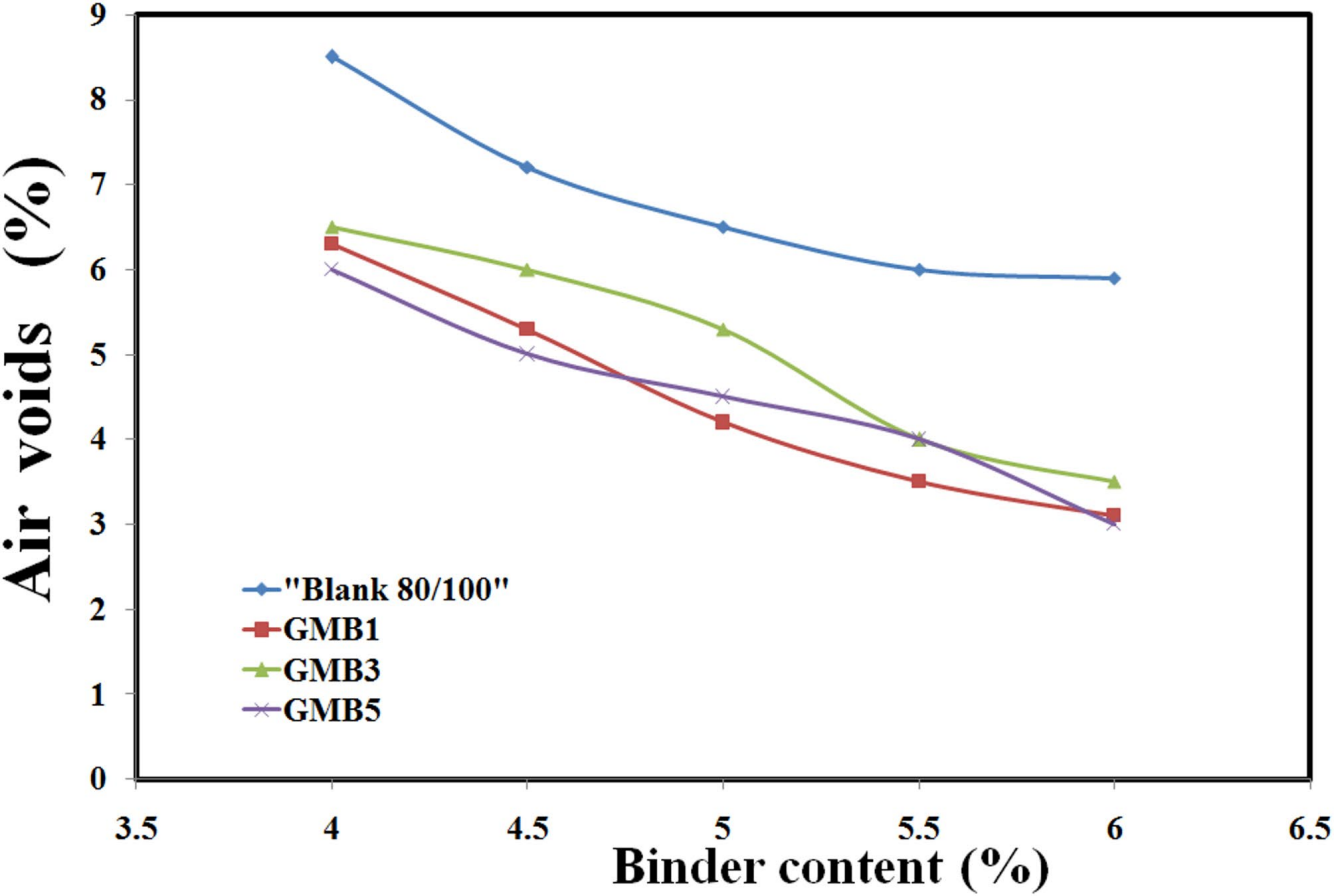
Air voids of GMB binders.

The prepared hot mix asphalt (HMA) mixtures, encompassing both the base binder (80/100 penetration grade) and the graphene-modified bitumen (GMB1, GMB3, and GMB5), were rigorously designed to meet the stringent requirements of ASTM D 6927 for surface courses subjected to high traffic volumes. The Marshall mix design method was employed to systematically determine the Optimal Binder Content (OBC) for each binder type, plotting key Marshall properties stability (kg), flow (mm), and air voids (%) against varying binder content, as illustrated in Figs. 14 and 15, and 16, respectively. For every blend, asphalt binder percentages ranged from 4% to 6% of the total solid materials by weight. The OBC for each mixture was precisely determined by identifying the binder content corresponding to the peak stability and an optimal air void percentage of 4%^61^.

A critical finding was that the base bitumen pen grade 80/100 proved unsuitable for the surface wearing course (4 C) when tested in the context of the HMA mixture. Its high softening properties led to all Marshall design results falling outside the specified range, underscoring its inadequacy for heavy traffic conditions. In stark contrast, the graphene-modified binders demonstrated high performance. The OBCs for GMB1, GMB3, and GMB5 were determined to be 5.26%, 5.33%, and 5.31% by total weight, respectively, indicating a consistent optimal binder requirement across the graphene-modified blends—the Marshall stability directly correlated with the HG-RGO content^62^, as depicted in Fig. 14.

Figure 14 illustrates the relationship between Marshall Stability and binder content (wt%) for both neat bitumen (Blank 80/100) and the graphene-modified bitumen mixtures (GMB1, GMB3, and GMB5). This analysis is paramount for assessing the asphalt mixture’s plastic flow resistance and overall structural integrity under traffic loading.

A consistent trend is observed across all binder types: Marshall stability increases with increasing binder content, reaching a peak (optimal stability), then decreases with further increases in binder content. This typical behaviour reflects the balance required in asphalt mixtures; insufficient binder leads to a lean, stiff mix with poor cohesion, while excessive binder causes a rich, tender mix prone to rutting and instability.

The Blank (80/100) bitumen mixture serves as the baseline, showing the lowest stability values across the entire range of binder content. Its peak stability is notably low, approximately 680 kg, and occurs around 5.0-5.2% binder content. This confirms the previously mentioned finding that this base bitumen is unsuitable for high-traffic surface courses, as its inherent softening properties lead to insufficient structural strength. Impact of HG-RGO Content on Stability: The most striking observation from the graph is the dramatic and consistent increase in Marshall Stability with the incorporation of HG-RGO, and further with higher HG-RGO content. GMB1 (1% HG-RGO) shows a significant improvement, reaching a peak stability of approximately 1,200 kg. This represents nearly an 80% increase in stability compared to the blank bitumen, even at the lowest graphene loading. At the same time, GMB3 (3% HG-RGO) demonstrates further enhancement, with its peak stability climbing to approximately 1,700 kg. However, GMB5 (5% HG-RGO) exhibits the most exceptional performance, achieving the highest stability of approximately 2,200 kg. This astounding value is over three times greater than that of the neat bitumen mixture, underscoring the profound reinforcing effect of HG-RGO.

Optimal Binder Content for Stability: Interestingly, while the magnitude of stability dramatically increases with graphene content, the Optimal Binder Content (OBC) corresponding to peak stability remains relatively consistent across all graphene-modified mixtures, clustering around 5.0% to 5.3% by weight. This suggests that the HG-RGO primarily enhances the inherent strength of the bitumen-aggregate mastic without significantly altering the optimal binder-to-aggregate ratio required for maximum mechanical interlocking^63^. The remarkable increase in Marshall stability with increasing HG-RGO content can be attributed to the unique properties of the highly graphitized graphene. HG-RGO, with its high mechanical strength, large specific surface area, and excellent interfacial adhesion with the bitumen matrix, forms a robust, three-dimensional reinforcing network within the binder^64^. This network effectively restricts the movement of bitumen molecules and increases the overall stiffness of the asphalt mixture. Under load, this graphene-reinforced mastic provides high resistance to plastic deformation, transferring stresses more efficiently across the aggregate skeleton^65^.

These findings have profound implications for pavement engineering. The significantly enhanced Marshall stability directly translates to improved rutting resistance and increased load-bearing capacity of asphalt pavements. Roads constructed with HG-RGO modified bitumen are thus expected to exhibit good durability, longer service life, and reduced maintenance requirements, especially in high-traffic areas or regions prone to high temperatures where conventional asphalt mixtures are more susceptible to permanent deformation. This study unequivocally demonstrates HG-RGO derived from coal-tar pitch as a highly effective and promising modifier for producing high-performance asphalt pavements^66^. Concurrently, the Marshall Flow test results (Fig. 15) showed statistically significant reductions in flow values for the HG-RGO modified mixtures compared to the base binder. GMB1, GMB3, and GMB5 exhibited flow values of 2.55 mm, 3.75 mm, and 3.54 mm, respectively (note: the relative order of these flows needs careful interpretation; if 2.55 is the lowest, compared to 3.75 for GMB3 and 3.54 for GMB5, it suggests an optimal content for flow reduction). These reduced flow values indicate greater resistance to plastic deformation under load, translating to good durability and less susceptibility to permanent deformation, particularly under hot conditions or heavy traffic^67^.

Furthermore, the air void percentage (Fig. 16) in the modified mixtures demonstrated a significant reduction compared to the base mixture, consistently achieving the desired 4% at their respective OBCs. This optimal air void content prevents premature ageing, improves durability, and contributes to rutting resistance. The combined improvements across stability, flow, and air voids unequivocally confirm the ability of HG-RGO to produce highly durable asphalt mixtures with high performance under demanding traffic conditions^68^.

#### Rutting test for asphalt mixtures

Using a double-wheel tracker, the permanent deformation response, specifically rutting susceptibility, was comprehensively evaluated for both the graphene-modified bitumen (GMB) mixtures and the control bitumen (80/100 penetration grade). All mixtures were meticulously prepared at their respective Optimal Binder Contents (OBCs), previously determined from the Marshall design results illustrated in Figs. 14 and 15, and 16. The control bitumen (80/100) was tested at its OBC of 5%, while each GMB sample was prepared at its corresponding OBC (e.g., GMB1 at 5.26%, GMB3 at 5.33%, GMB5 at 5.31%). The test was conducted under simulated harsh traffic and temperature conditions: a constant test temperature of 60 °C, a load of 705 N, and a total duration of 12,000 s (equivalent to 5,000 cycles at a rate of 25 cycles/minute).

**Fig. 17:**
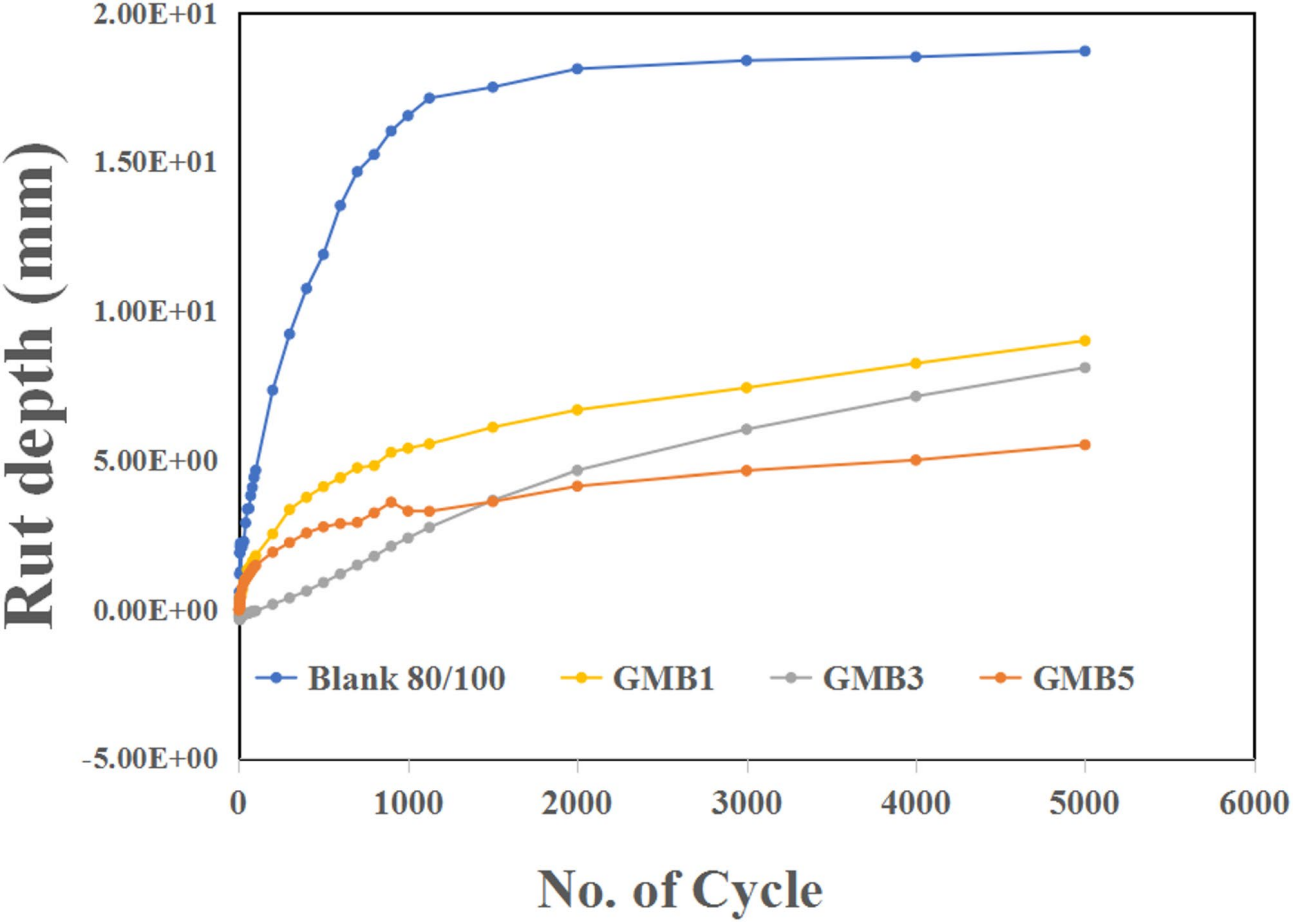
Rut depth for reference (Blank) sample 80/100 and GMB mixtures.

The rut depth plots in Fig. 17 graphically represent the mixtures’ resistance to permanent deformation. For the control bitumen (80/100 penetration grade), a catastrophic failure was observed almost immediately, with the sample showing complete failure within approximately 10 s of test initiation. This rapid and dramatic failure, characterized by the rut depth extending from near zero to around 18 mm as cycles progressed to 5,000, is directly attributed to the high viscous flow of the unmodified 80/100 bitumen between aggregate particles at elevated temperatures. This highlights its inherent vulnerability to rutting distress under demanding conditions, confirming its unsuitability for high-traffic wearing courses.

In stark contrast, the HG-RGO modified GMB mixtures demonstrated significantly enhanced rutting resistance. While the graph shows an unexpected increasing trend in rutting depth with higher graphene content (9 mm for GMB1, 8 mm for GMB3, and 5 mm for GMB5), this observation warrants further discussion in light of the otherwise high Marshall stability results. Although GMB5 shows the best rutting performance, all GMB mixtures still exhibited drastically reduced rut depths (approximately 5–9 mm) compared to the complete failure of the control sample, validating the positive impact of graphene modification. This reduction in rut depth, roughly half that of the control mix (consistent with previous findings by Akbari et al. ^59^, and Teshomeet al., ^69^. underscores the ability of HG-RGO to stiffen the bitumen matrix and improve its resistance to plastic flow. The improved rutting performance is fundamentally linked to the ability of the HG-RGO to create a strong reinforcing network within the bitumen, effectively restricting the movement of asphalt binder under repetitive loading and high temperatures. While an ideal scenario would show decreasing rut depth with increasing graphene content if stability is constantly increasing, the specific values obtained for GMB1, GMB3, and GMB5 might suggest an optimal graphene content for rutting performance that is not necessarily the highest stability content, or complex interactions at higher graphene loadings need further investigation to balance stiffness and other mix properties. Nonetheless, the overall trend unequivocally confirms that HG-RGO significantly mitigates permanent deformation in asphalt mixtures^70^.

Finally, indeed, all these findings can be explained in the light of the structure–property relationship between the produced filler, highly graphitized HG-RGO. Our results reveal that the distinctive microstructural characteristics of HG-RGO, high graphitisation degree (ID/IG = 0.3), few-layer stacking (4–5 layers), and extended lateral domains play a critical role in controlling its reinforcing role within the bituminous matrix. The high graphitic order ensures superior interfacial compatibility and high modulus due to higher structural order, which maximize the stress transfer efficiency. Besides the extended lateral dimensions, this means a high surface area, which in turn maximizes the interfacial adhesion and load transfer efficiency, translating into the observed enhancement in mechanical strength, rutting resistance, and thermal stability. Furthermore, the few-layer exfoliated configuration means no agglomeration, which also participates in maximizing the available interfacial area and ensuring uniform dispersion right through the binder phase. On the contrary, low graphitized or amorphous carbon materials, like carbon black or partially reduced graphene oxide, display weaker interfacial compatibility and lower stress transfer efficiency due to higher structural disorder, which limits the modulus value. Accordingly, these combined structural compensations distinguish HG-RGO from conventional carbon nanomaterials like carbon black or multi-walled carbon nanotubes, which typically demonstrate less surface activity and higher aggregation tendency, making HG-RGO a more efficient and stable modifier for bituminous systems. From another important point of view, the availability and cost-effectiveness of our facile one-pot hydrothermal strategy as compared with multi-step, time-consuming processes using strong acids and hazardous chemicals for humans and the environment lead to numerous structural defects, which in turn cause a decline in the output graphene structural characteristics and its unique properties. These findings collectively confirm that HG-RGO derived from coal-tar pitch can effectively prepare highly durable bitumen with good performance, offering a sustainable, cost-effective, and industrially viable solution for constructing long-lasting and resilient asphalt pavements.

## Conclusion

This investigation successfully demonstrated a novel, template-free, sole-step hydrothermal strategy for synthesizing Highly Graphitized Reduced Graphene Oxide (HG-RGO) directly from coal-tar pitch, an abundant and low-cost aromatic precursor. Comprehensive characterization (XRD, FTIR, Raman, HR-TEM, SAED) confirmed the high quality and graphitic nature of the synthesized HG-RGO. When incorporated into bitumen at various concentrations (GMB1, GMB3, GMB5), this HG-RGO significantly transformed the rheological and mechanical properties of the asphalt mixtures. The modified bitumen exhibited dramatically improved high-temperature performance and enhanced rutting resistance, overcoming the limitations of the base 80/100 bitumen, which proved unsuitable for high-traffic surface courses. Quantitatively, GMB binders have higher complex and lower loss modulus than base binders. The time sweep results able to expect that no change in the hardening of GMB samples will be occurred during or after service life., Marshall stability increased remarkably with higher HG-RGO content, reaching a peak of 2200 kg for GMB5, significantly outperforming the neat bitumen. Concurrently, the modified mixtures displayed reduced Marshall Flow values (e.g., 2.55 mm for GMB1), indicating high resistance to plastic deformation, and achieved optimal air void percentages (4%). These findings collectively confirm that HG-RGO derived from coal-tar pitch can effectively prepare highly durable bitumen with good performance, offering a sustainable, cost-effective, and industrially viable solution for constructing long-lasting and resilient asphalt pavements.

## Data Availability

The authors declare that the data supporting the findings of this study are available within the paper. Should any raw data files be needed in another format, they are available from the corresponding author upon reasonable request.
